# Effects of different exercise interventions on bone mineral density in elderly postmenopausal women: a network meta-analysis

**DOI:** 10.3389/fphys.2025.1633913

**Published:** 2025-09-25

**Authors:** Mingyu Ma, Wentao Su, Dansong Liu

**Affiliations:** School of Physical Education, Hubei University of Technology, Wuhan, Hubei, China

**Keywords:** mind-body training, multicomponent training, combined training, postmenopausal, bone mineral density, network meta-analysis

## Abstract

**Background:**

Various exercise interventions have been widely applied to enhance site-specific bone mineral density in menopausal females. This network meta-analysis aims to assess and compare the impact of these interventions on improving BMD in this demographic.

**Methods:**

A systematic search of PubMed, EMBASE, Cochrane Library, and Web of Science was made up to 4 December 2024 to detect randomized controlled trials (RCTs) comparing continuous endurance training whole-body vibration resistance training multicomponent training mind-body training intermittent training and combined training against control interventions. Primary outcomes included lumbar spine bone mineral density and femoral neck bone mineral density while secondary outcomes covered whole body bone mineral density and total hip bone mineral density A Bayesian random-effects NMA was performed.

**Results:**

Fifty-five RCTs involving 3,453 participants were included. Compared with the control group, MCT demonstrated greater efficacy in improving FNBMD (mean difference [MD] 0.02; 95% credible interval [CrI] [0.01, 0.04]). Based on the surface under the cumulative ranking (SUCRA), MBT ranked highest for LSBMD (75.9%), CT for WBBMD (77.6%), and MBT for THBMD (60.7%), suggesting potential benefits of these interventions.

**Conclusion:**

This study suggests that MBT, MCT, and CT may contribute to improving BMD in elderly postmenopausal women. However, further rigorously designed RCTs are warranted to validate these findings.

**Systematic Review Registration:**

https://www.crd.york.ac.uk/PROSPERO/, identifier CRD42025636067.

## 1 Background

With the growing aging population, the bone health of postmenopausal women has become a critical public health concern, particularly among the old population (Tartibian et al., 2011). The drop in estrogen levels during menopause catalyzes an accelerated process of bone resorption. This process ultimately leads to a marked depletion of bone mineral density (BMD) (Guadalupe-Grau et al., 2009). The resultant decline in BMD amplifies the susceptibility of osteoporotic fractures and associated complications, predisposing postmenopausal women to osteoporosis (OP) (Fu et al., 2011; De Aguiar et al., 2023). Notably, OP has been reported to impair functional ability and quality of life in this demographic (Ma et al., 2016; Benzinger et al., 2019; Autier et al., 2000), which is also strongly associated with elevated mortality rates, healthcare expenditures, and socioeconomic burdens on individuals, families, and society.

Currently, antiresorptive agents, especially bisphosphonates (BPPs), remain the mainstay pharmacological treatment for OP. However, due to their mechanism of binding to bone matrix and inhibiting bone resorption, they may also interfere with normal bone remodeling and reduce bone flexibility (Li et al., 2021; Russell et al., 2007). In contrast, exercise interventions have demonstrated promising benefits in improving BMD, preventing fractures, and mitigating OP progression in postmenopausal women, with minimal adverse effects (Tarantino et al., 2017; Kemmler et al., 2020). Accumulating evidence supports that exercise interventions, such as continuous endurance training (CET), resistance training (RT), mind-body training (MBT), whole-body vibration (WBV), combined training (CT), and multicomponent training (MCT), may effectively improve BMD in elderly postmenopausal women and further enhance their quality of life (de Oliveira et al., 2019; Aboarrage Junior et al., 2018; Marques et al., 2011a; Wen et al., 2017; Ammann and Rizzoli, 2003; Zou et al., 2017; Hejazi et al., 2022; Mohammad et al., 2020). Nevertheless, sustaining sufficient intensity and duration of exercise interventions may pose challenges for this demographic, making the identification of optimal exercise intervention essential (Burton et al., 2017; Sherrington et al., 2020).

Existing research on exercise interventions for BMD improvement in elderly postmenopausal females has produced a mosaic of inconsistent findings. For example, a meta-analysis by Hejazi et al. after incorporating 53 randomized controlled trials (RCTs) reported that RT, CT, and MBT significantly improved femoral neck bone mineral density (FNBMD), while CET and CT were effective in enhancing lumbar spine bone mineral density (LSBMD). Conversely, Mohammad Rahimi et al. (Mohammad et al., 2020) observed no significant effects of CET on LSBMD or FNBMD. Notably, WBV appeared to outperform CET, RT, and CT in improving LSBMD (Mohammad et al., 2020). Additionally, the analysis of Zehnacker et al. on 20 RCTs (Zehnacker and Bemis-Dougherty, 2007) further highlighted that site-specific high-intensity weight-bearing training improved BMD in the spine and hip among menopausal females (Zehnacker and Bemis-Dougherty, 2007). These discrepancies may be ascribed to variations in exercise interventions, intervention durations, intensities, demographic characteristics, and study designs. Furthermore, the scarcity of high-quality trials directly comparing different exercise interventions limits definitive conclusions regarding the most efficacious intervention (Hejazi et al., 2022). To date, systematic reviews and meta-analyses consistently report that exercise interventions help mitigate the loss of BMD in elderly postmenopausal women. However, the relative efficacy of various exercise interventions across demographics with osteoporosis, osteopenia, or normal bone mass remains unclear.

Network meta-analysis (NMA) facilitates the concurrent comparison of multiple exercise interventions, even when direct head-to-head trials are unavailable. Moreover, NMA allows for the ranking of interventions by efficacy outcome, thereby offering comprehensive evidence to inform clinical decision-making. Therefore, the objective of systematic review and NMA in this study is to elucidate the impacts of diverse exercise interventions on BMD among elderly postmenopausal women by synthesizing comparative evidence either directly or indirectly. The findings will provide scientifically grounded recommendations for designing exercise strategies specifically tailored to improve BMD in this demographic.

## 2 Methods

### 2.1 Design and registration

The NMA adhered to the Preferred Reporting Items for Systematic Reviews and Meta-Analyses (PRISMA) Guidelines. The study protocol was prospectively registered in the International Prospective Register of Systematic Reviews (PROSPERO; Registration No. CRD42025636067).

### 2.2 Inclusion and exclusion criteria

The inclusion criteria were defined using the PICOS framework (Participants, Interventions, Comparators, Outcomes, Study Design) as follows: (1) Participants: Postmenopausal women aged ≥60 years with non-adherence to exercise guidelines (defined as engaging in less than 120 min of weekly physical activity). (2) Interventions: At least one form of exercise intervention (regardless of form or duration), including CET, RT, CT, WBV, INT, MBT, or MCT. These exercise interventions are among the most extensively investigated modalities for improving BMD in older postmenopausal women (de Oliveira et al., 2019; Aboarrage Junior et al., 2018; Marques et al., 2011a; Wang et al., 2015; Li et al., 2023; Riaz et al., 2024). (3) Comparator: Non-exercise control groups. (4) Outcomes: At least one of the following outcomes: Primary outcomes included LSBMD and FNBMD, while secondary outcomes covered whole body bone mineral density (WBBMD) and total hip bone mineral density (THBMD). (5) Study Design: Randomized controlled trials (RCTs) only. (6) Language: Studies published solely in English.

The exclusion criteria comprised: (1) Non-postmenopausal cohorts; (2) Studies with undefined intervention protocols; (3) Non-RCT designs, such as cohort studies, review articles, case reports, descriptive studies, opinion pieces, or conference abstracts; (4) Studies with incomplete, inaccurate, or irretrievable data.

### 2.3 Search strategy

Two investigators (M.M.Y. and L.D.S.) independently performed a comprehensive literature search in PubMed, Embase, the Cochrane Central Register of Controlled Trials (CENTRAL), and Web of Science up to 4 December 2024. No restrictions were imposed regarding publication type, date, or status. The search combined Medical Subject Headings (MeSH) and free-text keywords, covering all known variants (postmenopausal) AND (exercise OR physical activity OR continuous endurance training OR mind–body training OR intermittent training OR resistance training OR multicomponent training OR combined training) AND (RCT). To minimize the risk of missing eligible studies, reference lists of relevant articles were also manually screened by both investigators. (The full search strategy is detailed in Supplementary Appendix A).

### 2.4 Study selection

Using the predefined inclusion and exclusion criteria, two investigators (M.M.Y and L.D.S) independently screened studies. All potentially relevant records were input into EndNote X9, with duplicates removed. Title/abstract screening was made to exclude irrelevant studies, followed by full-text evaluations. Discrepancies were settled via discussion or consultation with a third investigator (S.W.T).

### 2.5 Data extraction and quality assessment

The information was gathered via a standardized data extraction form as follows: (1) Study characteristics, including the first author’s name, publication year, and country; (2) Participant characteristics, including age, sample size of intervention and control groups, and demographic type (healthy vs osteoporotic); (3) Intervention details, including exercise intervention measures and duration; (4) Reported outcomes. Data were extracted by one investigator (M.M.Y) and cross-validated by another investigator (L.D.S).

Two investigators (M.M.Y. and L.D.S.) independently examined the potential risk of bias for every included study via the Cochrane Risk of Bias 2.0 (ROB 2.0) tool (Sterne et al., 2019; McGuinness and Higgins, 2021). Any discrepancies were settled via discussion. The tool evaluates six domains: (1) Randomization process; (2) Deviations from intended interventions; (3) Missing outcome data; (4) Measurement of the outcome; (5) Selection of the reported result; (6) Overall bias. Each domain was assigned one of three ratings: “low risk,” “some concerns,” or “high risk.” The overall bias classification followed a hierarchical structure: “low risk” (all domains rated as low), “some concerns” (at least one domain with some concerns but none with high risk), or “high risk” (at least one domain rated as high risk). Discrepancies were resolved via discussion or, if necessary, consultation with a third investigator.

### 2.6 Data synthesis and statistical analysis

Statistical models employing Bayesian frameworks were built via JAGS software (gemtc 0.8-2 and rjags 4–10 packages) in R (version 4.4.3). Continuous outcomes were analyzed using mean differences (MDs) with 95% credible interval (CrI) to quantify effect sizes. Random-effects models addressed clinical heterogeneity across studies (e.g., country, exercise interventions, participant health status, intervention duration, calcium supplementation) throughout NMA. Four Markov Chain Monte Carlo (MCMC) chains were run for each outcome, with 50,000 iterations per chain, discarding the first 20,000 iterations as burn-in. Convergence was evaluated via trace plots and the Gelman–Rubin–Brooks diagnostic statistic (Brooks and Gelman, 1998). Relative intervention rankings by outcome were estimated through surface under the cumulative ranking (SUCRA) (Veron et al., 2016). Higher SUCRA values mean better intervention rankings. Model fit and consistency were assessed by comparing the Deviance Information Criterion (DIC). A DIC difference of less than 5 was considered indicative of good consistency, in which case the consistency model was retained (Dempster, 1997). Local inconsistencies in closed loops were analyzed via node-splitting. The presence of publication bias was systematically explored using adjusted funnel plots. Network plots and adjusted funnel plots were generated using Stata version 15.0.

## 3 Results

### 3.1 Search results

**FIGURE 1:**
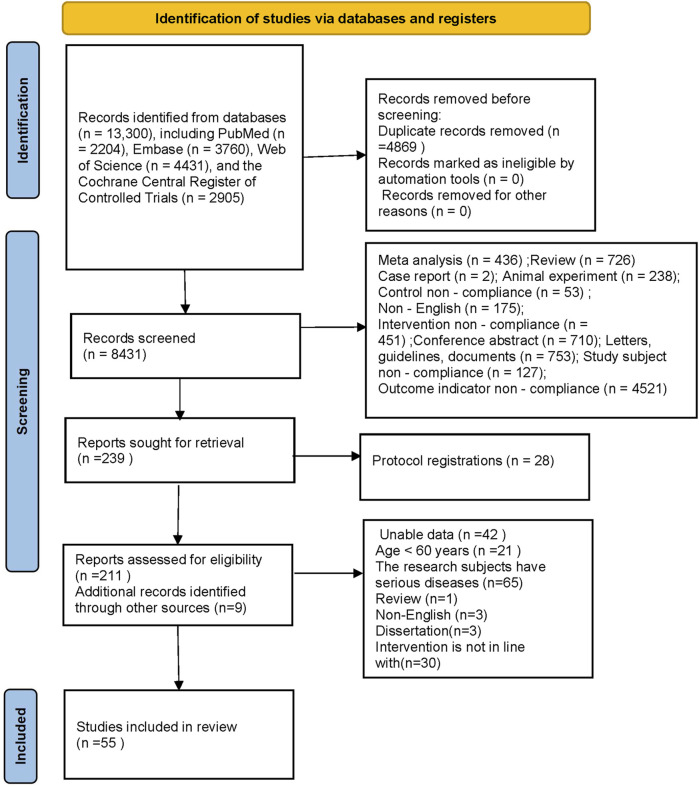
PRISMA flow diagram demonstrating the study selection process for the network.

The PRISMA flow diagram (Figure 1) details the study selection process. The initial search identified 13,300 records from the four databases. Following the removal of 4,869 duplicates, 8,192 studies were excluded via title/abstract screening. Full-text reviews excluded 193 studies (non-RCT designs, irrelevant populations, or incomplete data) (details provided in Figure 1). An additional 9 records were identified through reference screening, resulting in 55 eligible studies eventually incorporated into the NMA.

### 3.2 Characteristics of included studies

Table 1 sets out the characteristics and detailed information of the studies incorporated into the NMA. Between 1998 and 2024, fifty-five RCTs fulfilled the inclusion criteria. Geographically, seventeen studies were conducted in the Americas (de Oliveira et al., 2019; Aboarrage Junior et al., 2018; Chubak et al., 2006; Moreira et al., 2014; Bocalini et al., 2010; Bocalini et al., 2009; Martin and Notelovitz, 1993; Bloomfield et al., 1993; Smidt et al., 1992; Grove and Londeree, 1992; Slatkovska et al., 2011; Jessup et al., 2003; Brentano et al., 2008; Dalsky et al., 1988; Chuin et al., 2009; Pruitt et al., 1995; Rhodes et al., 2000), seventeen in Asia (Tartibian et al., 2011; Wen et al., 2017; Wang et al., 2015; Li et al., 2023; Riaz et al., 2024; Abdul-Al et al., 2024; Brooke-Wavell et al., 1997; Lai et al., 2013; Song and Yang, 2021; Kwon et al., 2008; Iwamoto et al., 2001; Iwamoto et al., 1998; Ruan et al., 2008; Yamazaki et al., 2004; Yu et al., 2019; Nambi et al., 2020; Park et al., 2008), fifteen in Europe (Marques et al., 2011a; Jamka et al., 2021; Hartley et al., 2020; Marín-Cascales et al., 2019; Marin-Cascales et al., 2015; Brooke-Wavell et al., 2001; von Stengel et al., 2011; Verschueren et al., 2004; Englund et al., 2005; Marques et al., 2011b; Kemmler et al., 2010; Korpelainen et al., 2006; Mosti et al., 2013; Tolomio et al., 2010; Santin-Medeiros et al., 2015), five in Oceania (Nicholson et al., 2015; Young et al., 2007; Watson et al., 2018; Beck and Norling, 2010; Lord et al., 1996), and one in Africa (ElDeeb and Abdel-Aziem, 2020). Collectively, these RCTs encompassed 3,453 menopause women aged between 54.1 and 82.3 years, with sample sizes between 14 and 227 participants. Among the participants, 2,292 were healthy, 258 were classified as overweight or obese, and 903 were diagnosed with osteoporosis or osteopenia. The interventions varied across studies: seventeen studies involved CET (Aboarrage Junior et al., 2018; Wen et al., 2017; Moreira et al., 2014; Martin and Notelovitz, 1993; Bloomfield et al., 1993; Grove and Londeree, 1992; Brooke-Wavell et al., 1997; Iwamoto et al., 2001; Iwamoto et al., 1998; Yamazaki et al., 2004; Yu et al., 2019; Nambi et al., 2020; Jamka et al., 2021; Brooke-Wavell et al., 2001; Marques et al., 2011b; Kemmler et al., 2010; Tartibian et al., 2011), fourteen studies focused on RT (Chubak et al., 2006; Bocalini et al., 2010; Bocalini et al., 2009; Smidt et al., 1992; Brentano et al., 2008; Chuin et al., 2009; Pruitt et al., 1995; Rhodes et al., 2000; Abdul-Al et al., 2024; Verschueren et al., 2004; Marques et al., 2011b; Mosti et al., 2013; Nicholson et al., 2015; Watson et al., 2018), sixteen studies implemented MCT (Marques et al., 2011a; Chubak et al., 2006; Jessup et al., 2003; Dalsky et al., 1988; Lai et al., 2013; Kwon et al., 2008; Park et al., 2008; Marín-Cascales et al., 2019; Marin-Cascales et al., 2015; von Stengel et al., 2011; Englund et al., 2005; Kemmler et al., 2010; Korpelainen et al., 2006; Tolomio et al., 2010; Nicholson et al., 2015; Lord et al., 1996), twelve studies utilized WBV (de Oliveira et al., 2019; Slatkovska et al., 2011; Lai et al., 2013; Song and Yang, 2021; Ruan et al., 2008; Marín-Cascales et al., 2019; Marin-Cascales et al., 2015; von Stengel et al., 2011; Verschueren et al., 2004; Santin-Medeiros et al., 2015; Beck and Norling, 2010; ElDeeb and Abdel-Aziem, 2020), five studies employed CT (Wang et al., 2015; Li et al., 2023; Grove and Londeree, 1992; Jamka et al., 2021; Young et al., 2007), six studies incorporated MBT (de Oliveira et al., 2019; Wang et al., 2015; Riaz et al., 2024; Nambi et al., 2020; Korpelainen et al., 2006; Young et al., 2007), two studies utilized high-intensity INT (Smidt et al., 1992; Hartley et al., 2020). Intervention protocols are detailed in Supplementary Table S1.

**TABLE 1:** Characteristics of studies included in the NMA.

First author	Year of publication	Country	Sample size		Age (mean ± SD)		Population		Intervention	Outcomes
			Intervention group	Control group	Intervention group	Control group	Type	Intervention group	Control group	
Abdul	2024	Lebanon	32	17	65.3 ± 3.42	65.47 ± 2.52	Health	RT	Con	A, B, C, D
Bocalini	2010	Brazil	13	12	66 ± 9	64 ± 8	Health	RT	Con	B, C
Bocalini	2009	Brazil	15	10	69 ± 9	67 ± 8	Health	RT	Con	B, C
Bloomfield	1993	United States	7	7	62.0 ± 0.8	58.8 ± 3.6	Health	CET	Con	B, C
BRENTANO	2008	Brazil	19	9	64.8	64.8	Osteoporosis	RT	Con	C
Beck	2010	Australia	28	14	68.6 ± 8.2	74.2 ± 8.1	Health	WBV	Con	A, B, C
CHUBAK	2006	United States	87	86	60.7 ± 6.7	60.6 ± 6.8	Obesity	MCT	RT	A
Cascales	2019	Spain	15	13 10	59.6 ± 5.9	58.0 ± 7.3 62.4 ± 5.1	Health	WBV	MCT Con	B, C
Cascales	2015	Spain	14	14 10	60.1 ± 5.8	57.7 ± 7.1 62.4 ± 5.1	Health	WBV	MCT Con	A
Chuin	2009	Canada	11	7	65.4 ± 3.5	67.4 ± 3.8	Health	RT	Con	B, C
Dalsky	1988	United States	11	14	61.6 ± 1.0	62.6 ± 1.2	Health	MCT	Con	B
ElDeeb	2019	Egypt	22	21	55.09 ± 4.19	57.29 ± 4.44	Osteoporosis	WBV	Con	B, C
Englund	2005	Sweden	21	19	72.8 ± 3.6	73.2 ± 4.9	Health	MCT	Con	A, B, C
GROVE	1992	United States	5	5	54.0 ± 1.9	56.6 ± 4.3	Health	CT	CET	A
Hartley	2020	United Kingdom	35	35	61.7 ± 4.3	61.7 ± 4.3	Health	INT	Con	B, C
Iwamoto	2001	Japan	8	20	65.3 ± 4.7	64.9 ± 5.7	Osteoporosis	CET	Con	B
Iwamoto	1998	Japan	15	20	64.8 ± 6.1	64.8 ± 5.7	Osteoporosis	CET	Con	B
Jamka	2021	Poland	44	41	55 ± 7	55 ± 7	Obesity	CET	CT	A, B, C
Junior	2018	Brazil	15	10	65 ± 7	65 ± 7	Health	CET	Con	A, B
Jessup	2003	United States	9	9	69.1 ± 2.8	69.4 ± 4.2	Health	MCT	Con	B, C
Kemmler	2010	Germany	112	115	69.2 (4.1)	68.9 (3.9)	Health	CET	Con	B, C
Kwon	2008	South Korea	20	20	77.4 ± 2.56	77.0 ± 3.33	Health	MCT	Con	A, B, C
Korpelainen	2006	Finland	84	76	72.9	72.8	Osteoporosis	MBT	Con	C
lai	2013	China	14	14	60.1 ± 7.1	62.4 ± 7.1	Health	WBV	Con	B
Li	2023	China	16	16	63.11 ± 4.59	62.47 ± 4.79	Osteoporosis	CT	Con	A, B
Lord	1996	Australia	68	70	71.7 ± 5.4	71.5 ± 5.3	Health	MCT	Con	B, C
Moreira	2014	Brazil	59	41	58.6 ± 6.71	59.3 ± 6.07	Health	CET	Con	A, B, C
MARTIN	1993	United States	36	19	54.5 ± 8.3	56.7 ± 6.9	Health	CET	Con	B
Marques	2011	Portugal	15	19	67.3 ± 5.2	70.3 ± 5.5	Health	RT	CET	C, D
Marques	2011	Portugal	30	30	70.1 ± 5.4	68.2 ± 5.7	Health	MCT	Con	B, C
MOSTI	2013	Norway	8	8	61.9 ± 5.0	66.7 ± 7.4	Osteoporosis	RT	Con	B, C, D
Nicholson	2014	Australia	24	26	66.0 ± 4.1	65.6 ± 4.7	Health	RT	Con	A, B, C, D
Nambi	2020	Saudi Arabia	15	15	56.3 ± 2.5	57.6 ± 2.7	Osteoporosis	MBT	CET	A
Oliveira	2018	Brazil	17	17 17	56.4 (6.5)	55.6 (6.8) 54.1 (5.3)	Health	WBV	MBT Con	B, C, D
Park	2008	Japan	25	25	68.3 ± 3.6	68.4 ± 3.4	Health	MCT	Con	B, C
Pruitt	1995	United States	7	11	67.6 ± 1.4	69.6 ± 4.2	Health	RT	Con	B, C, D
Riaz	2024	Pakistan	22	21	58.3 ± 5.1	58.0 ± 5.5	Osteoporosis	MBT	Con	B, C
Rhodes	2000	Canada	20	18	68.8 ± 3.2	68.2 ± 3.5	Health	RT	Con	B, C
SMIDT	1992	United States	22	27	56.6 ± 6.6	55.4 ± 8.0	Health	RT	Con	B
Slatkovska	2011	Canada	135	67	60.1 ± 6.5	60.8 ± 5.5	Health	WBV	Con	B, C, D
Song	2021	China	56	20	64.0 ± 2.1	64.2 ± 1.8	Health	WBV	Con	B, C, D
Stengel	2011	Germany	50	50	68.8 ± 3.6	68.6 ± 3.0	Health	WBV	MCT	B, D
Santin	2015	Spain	19	18	82.3 ± 5.1	82.2 ± 6.4	Health	WBV	Con	C, D
Tolomio	2010	Italy	58	67	62 ± 5.0	64 ± 5.3	Osteoporosis	MCT	Con	A, C
Tartibian	2011	Iran	20	18	61.4 ± 6.9	58.9 ± 8.1	Health	CET	Con	B, C
Verschueren	2004	Belgium	25	22	64.6 ± 3.3	63.90 ± 3.8	Health	WBV	RT	A, B
Wavell	2001	United Kingdom	20	16	65.0 ± 3.1	65.5 ± 2.7	Health	CET	Con	B, C
WAVELL	1997	Japan	38	40	64.9 ± 3.0	64.2 ± 3.1	Health	CET	Con	C
Wen	2016	China	24	22	57.5 ± 3.5	58.8 ± 3.2	Osteoporosis	CET	Con	A, D
Watson	2018	Australia	43	43	65 ± 5	65 ± 5	Osteoporosis	RT	Con	B, C
Wang	2015	China	34	37 35	58.54 ± 3.37	57.93 ± 3.22 58.54 ± 3.37	Health	MBT	CT Con	C
Xiang_yan	2008	China	51	43	61.23 ± 8.20	63.73 ± 5.45	Osteoporosis	WBV	Con	B, C
Young	2007	Australia	15	15 15	61.9 ± 7.0	61.5 ± 5.2 64.4 ± 8.0	Health	CET	CT MBT	B
Yamazaki	2004	Japan	27	15	64.2 ± 2.9	65.7 ± 2.7	Osteoporosis	CET	Con	B
Yu	2019	China	40	40	61.5 ± 7.5	62.5 ± 6.6	Osteoporosis	CET	Con	C

Outcome Measures: A: Whole-body BMD; B: Lumbar Spine BMD; C: Femoral Neck BMD; D: Total Hip BMD; Intervention Abbreviations: MCT: multicomponent training; MBT: Mind-Body Training; WBV: Whole-Body Vibration; RT: resistance training; CET: continuous endurance training; CT: Combined Training (Aerobic + Resistance); INT: intermittent training.

### 3.3 Quality assessment

**FIGURE 2:**
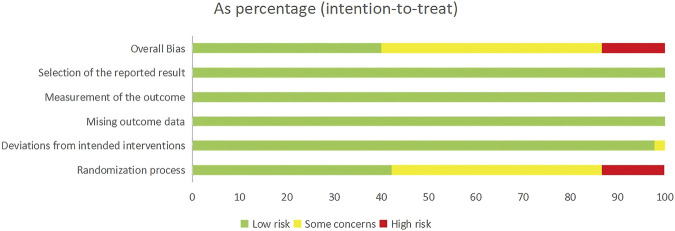
Risk of bias assessments for included RCTs.

The risk of bias across the included RCTs was examined via standardized criteria. Out of the fifty-five studies, twenty-five (45.5%) were assessed as having some concerns regarding bias, twenty-three (41.8%) were deemed low risk, and seven (12.7%) were considered high risk. Most RCTs adequately described their randomization procedures. However, the primary sources of bias stemmed from insufficient reporting on allocation concealment and blinding of outcome assessors. A comprehensive risk of bias assessment for every study is available in Supplementary Figure S1. Figure 2 sums up the risk of bias assessments.

### 3.4 NMA

#### 3.4.1 Primary outcomes

##### 3.4.1.1 LSBMD

**FIGURE 3:**
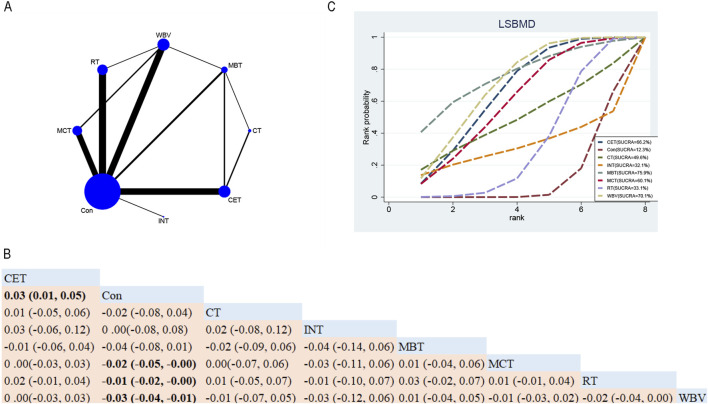
Network Diagram and NMA Results. **(A)** Network diagram for LSBMD (g/cm^2^). **(B)** Relative efficacy of diverse exercise interventions on LSBMD. Note: Estimates are expressed as MD with 95% CrI (in parentheses). Read comparisons between interventions from left to right. Effectiveness estimates are at the intersection of exercise intervention columns and rows. Significant results are shown in bold. **(C)** SUCRA for different exercise interventions on LSBMD. The exercise interventions in the plot are: MCT–Multicomponent training, MBT–Mind-body training, WBV–Whole-body vibration, RT–Resistance training, CET–Continuous endurance training, CT–Combined training, INT–Intermittent training, Con–Control group.

A total of forty-one RCTs evaluated the effects of seven exercise interventions on LSBMD in postmenopausal women. NMA results (Figure 3A) revealed that, compared to the control group, WBV (MD: 0.03; 95% CrI [0.01, 0.04]), MCT (MD: 0.02; 95% CrI [0.00, 0.05]), and RT (MD: 0.01; 95% CrI [0.00, 0.02]) significantly increased LSBMD. Conversely, CET (MD: 0.03; 95% CrI [0.01, 0.05]) was associated with a significant increase in LSBMD (Figure 3B). SUCRA analysis indicated that MBT ranked highest for enhancing LSBMD (75.9%), suggesting it may be a particularly promising intervention (Figure 3C).

##### 3.4.1.2 FNBMD

**FIGURE 4:**
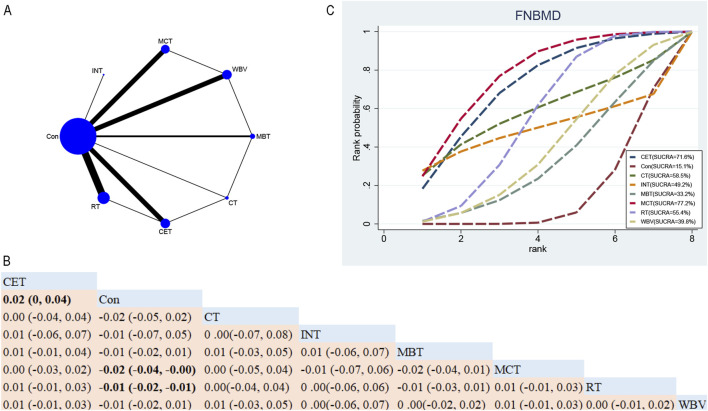
Network Diagram and NMA Results. **(A)** Network diagram for FNBMD (g/cm^2^). **(B)** Relative effects of exercise interventions on FNBMD. Note: Estimates are presented as MD with 95% CrI (in parentheses). Read comparisons between interventions from left to right. Effectiveness estimates are at the intersection of exercise intervention columns and rows. Significant results are shown in bold. Continuous Endurance Training; Multicomponent Training; Resistance Training. **(C)** SUCRA for different exercise interventions on FNBMD. The exercise interventions in the plot are: MCT–Multicomponent training, MBT–Mind-body training, WBV–Whole-body vibration, RT–Resistance training, CET–Continuous endurance training, CT–Combined training, INT–Intermittent training, Con–Control group.

Overall, thirty-eight RCTs assessed the impacts of seven exercise interventions on FNBMD in postmenopausal women (Figure 4A). NMA results (Figure 4B) showed that compared with the control group, MCT (MD 0.02; 95% CrI [0.00, 0.04]) and RT (MD 0.01; 95% CrI [0.01, 0.02]) significantly increased FNBMD (Figure 4B). In contrast, compared to CET, the control group (MD 0.02; 95% CrI [0.00, 0.04]) indicated that CET significantly reduced FNBMD in postmenopausal women (Figure 4B). Based on SUCRA, MCT achieved the highest ranking for improving FNBMD (77.3%), supporting its potential as the most effective intervention (Figure 4C).

#### 3.4.2 Secondary outcomes

##### 3.4.2.1 WBBMD

**FIGURE 5:**
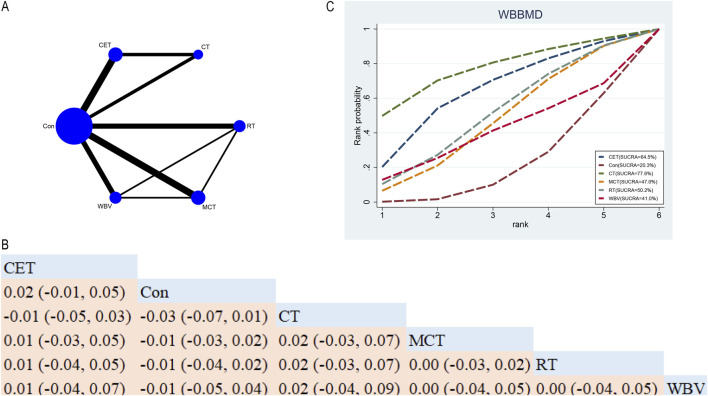
Network Diagram and NMA Results **(A)** Network diagram for WBBMD (g/cm^2^). **(B)** Relative effects of different exercise interventions on WBBMD. Note: Estimates are presented as MD with 95% CrI (in parentheses). Read comparisons between interventions from left to right. Effectiveness estimates are at the intersection of exercise intervention columns and rows. Significant results are shown in bold. **(C)** SUCRA for different exercise interventions on WBBMD. The exercise interventions in the plot are: MCT–Multicomponent training, WBV–Whole-body vibration, RT–Resistance training, CET–Continuous endurance training, CT–Combined training, Con–Control group.

In total, fifteen RCTs explored the efficacy of five exercise interventions on WBBMD in postmenopausal women (Figure 5A). NMA results (Figure 5B) revealed no statistically significant disparities among the different exercise interventions (Figure 5B). SUCRA identified CT as the top-ranked intervention for WBBMD (77.6%), indicating its strong potential efficacy (see Figure 5C).

##### 3.4.2.2 THBMD

**FIGURE 6:**
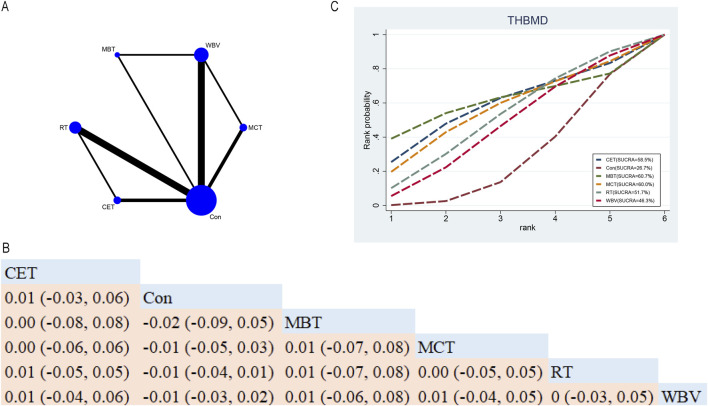
Network Diagram and NMA Results. **(A)** Network diagram for THBMD (g/cm^2^). **(B)** Relative effects of different exercise interventions on total hip bone mineral density (THBMD). Note: Estimates are presented as MD with 95% CrI (in parentheses). Read comparisons between interventions from left to right. Effectiveness estimates are at the intersection of exercise intervention columns and rows. Significant results are shown in bold. **(C)** SUCRA for different exercise interventions on THBMD. The exercise interventions in the plot are: MCT–Multicomponent training, MBT–Mind-body training, WBV–Whole-body vibration, RT–Resistance training, CET–Continuous endurance training, Con–Control group.

In total, ten RCTs probed into the roles of five exercise interventions on THBMD in postmenopausal older women (Figure 6A). NMA results (Figure 6B) demonstrated that no statistically significant differences were found among the various exercise interventions (Figure 6B). SUCRA indicated that MBT ranked highest for increasing THBMD (60.7%), suggesting it may be a promising intervention (see Figure 6C).

### 3.5 Subgroup and sensitivity analyses

Only LSBMD and FNBMD met the criteria for subgroup analyses. For LSBMD, stratification by health status revealed that CET was significantly effective in healthy participants (MD 0.03; 95% CrI [0, 0.07]), whereas WBV was significantly effective in those with osteoporosis (MD 0.05; 95% CrI [0.02, 0.09]). SUCRA rankings also identified CET (78.9%) and WBV (81.0%) as top interventions. This suggests that exercise interventions exert beneficial effects across different health statuses, although the optimal modality may vary. For FNBMD, CET (MD 0.02; 95% CrI [0, 0.04]) and MCT (MD 0.03; 95% CrI [0.01, 0.05]) were both significantly effective in healthy participants. No statistically significant differences were observed in osteoporotic participants, but SUCRA rankings placed MCT (79.2%) and WBV (76.4%) at the top. Notably, the ranking of WBV differed substantially from the overall results, highlighting the need for further validation with high-quality RCTs (see Supplementary Figure S2A–C, S3A–C, S4A–C,S5A–C).

Due to the limited number of eligible studies, subgroup analyses were not performed for WBBMD and THBMD; instead, sensitivity analyses were conducted. For WBBMD, exclusion of osteoporotic participants revealed no great differences among interventions, although SUCRA ranking placed CT (67.6%) as the leading intervention, consistent with the overall findings. This implies that health status has only a modest influence on whole-body bone mass. For THBMD, exclusion of osteoporotic participants likewise revealed no significant differences, but SUCRA rankings positioned MBT (61.8%) at the top, again aligning with the overall results. These findings imply that health status may have little impact on hip bone density, though the conclusions remain constrained by the limited evidence base. In summary, the results for WBBMD and THBMD should be interpreted cautiously, and additional high-quality RCTs are needed to substantiate these observations. (See Supplementary Figure S6A–C, S7A–C).

### 3.6 Consistency and publication bias assessment

DIC served as a tool to compare consistency and inconsistency models. The variation in DIC values across all closed-loop models was lower than 5, indicating good model consistency with DIC. Node-splitting analysis was performed to assess and explore local inconsistencies in the closed loops, and the results showed no evidence of local inconsistency, as listed in Table 2. Regarding publication bias, adjusted funnel plots (Supplementary Figures S8-S17) showed no traces of publication bias.

**TABLE 2:** Local inconsistency evaluation and exploration for closed-loop results using node splitting method.

Outcomes	Comparison	Direct	Indirect	Network	P.Value CrI
Lumbar spine	Con vs CET	−0.03 (−0.05,-0.01)	−0.04 (−0.18, 0.11)	−0.03 (−0.05,-0.01)	0.86
Lumbar spine	MBT vs CET	0.02 (−0.05, 0.10)	−0.01 (−0.08, 0.05)	0.01 (−0.04, 0.06)	0.51
Lumbar spine	MBT vs Con	0.04 (−0.01, 0.10)	0.01 (−0.11, 0.14)	0.04 (−0.01, 0.09)	0.69
Lumbar spine	MBT vs CT	−0.01 (−0.18, 0.14)	0.03 (−0.05, 0.11)	0.02 (−0.05, 0.09)	0.67
Lumbar spine	WBV vs MBT	0.01 (−0.09, 0.12)	−0.02 (−0.07,0.04)	−0.01 (−0.06, 0.05)	0.64
Lumbar spine	WBV vs MCT	−0.00 (−0.06,0.06)	0.01 (−0.02, 0.03)	0.01 (-0.02.0.03)	0.85
Lumbar spine	WBV vs RT	−0.01 (-0.09,0.08)	0.02 (−0.00,0.04)	0.02 (−0.00,0.04)	0.52
Femoral neck	Con vs CET	−0.02 (−0.04,-0.00)	−0.01 (−0.09,0.06)	−0.02 (−0.04,0.00)	0.85
Femoral neck	CT vs CET	−0.00 (−0.05,0.05)	−0.00 (−0.07,0.05)	−0.00 (−0.04, 0.04)	0.89
Femoral neck	RT vs CET	−0.01 (−0.08, 0.05)	−0.01 (−0.03, 0.01)	−0.01 (−0.03,0.01)	0.86
Femoral neck	CT vs Con	0.02 (−0.03.0.07)	0.02 (−0.04, 0.08)	0.01 (−0.02,0.05)	0.94
Femoral neck	MBT vs CT	0.00 (−0.07, 0.06)	−0.01 (−0.08,0.05)	−0.01 (-0.05,0.03)	0.76
Femoral neck	WBV vs MBT	0.00 (−0.07, 0.08)	0.00 (−0.02, 0.03)	0.00 (−0.02, 0.03)	0.98
Femoral neck	WBV vs MCT	0.01 (−0.12,0.10)	0.01 (−0.04,0.01)	−0.01 (−0.03,0.01)	0.98
Whole body	Con vs CET	−0.02 (−0.06,0.02)	−0.02 (−0.11, 0.06)	−0.02 (−0.05, 0.01)	0.89
Whole body	CT vs CET	0.01 (-0.05,0.06)	0.01 (−0.06,0.08)	0.01 (−0.03, 0.05)	0.89
Whole body	CT vs Con	0.04 (−0.02, 0.09)	0.02 (−0.05, 0.09)	0.03 (−0.01, 0.07)	0.70
Whole body	MCT vs Con	0.01 (−0.03,0.04)	0.02 (−0.04 0.08)	0.01 (−0.02, 0.03)	0.64
Whole body	RT vs Con	0.02 (−0.02, 0.06)	0.00 (−0.05, 0.06)	0.01 (−0.02, 0.04)	0.62
Whole body	RT vs MCT	−0.00 (−0.04,0.04)	0.01 (−0.04, 0.06)	0.00 (−0.02,0.03)	0.68
Whole body	WBV vs MCT	−0.01 (-0.09,0.08)	0.00 (−0.06,0.06)	−0.00 (−0.05.0.04)	0.93
Whole body	WBV vs RT	0.00 (−0.06, 0.07)	−0.02 (−0.10,0.06)	−0.00 (−0.05,0.04)	0.66
Total hip	RT vs CET	0.01 (−0.08, 0.11)	−0.01 (−0.08,0.06)	−0.01 (−0.05, 0.05)	0.67
Total hip	WBV vs MCT	−0.00 (−0.06,0.06)	−0.02 (−0.10,0.06)	−0.01 (−0.05, 0.04)	0.70

## 4 Discussion

### 4.1 Key findings

A comprehensive literature search identified 55 RCTs involving 3,454 participants, and a Bayesian NMA was conducted to evaluate the comparative effects of seven exercise interventions—namely CET, RT, MCT, MBT, WBV, INT, and CT—on BMD in postmenopausal women. Outcomes were analyzed across multiple skeletal sites, including the lumbar spine, femoral neck, total hip, and whole body. The results suggested MBT as a promising intervention for attenuating the loss of LSBMD, while MCT appeared potentially beneficial for improving FNBMD. In contrast, CET, CT, and INT showed no significant effects on the primary outcomes of BMD. For the secondary outcomes, SUCRA analysis ranked CT highest for WBBMD and MBT for THBMD, implying relative advantages of these modalities, although no definitive statistical superiority was established.

#### 4.1.1 Effects of different exercise interventions on primary BMD outcomes

##### 4.1.1.1 LSBMD

NMA revealed that MBT, primarily consisting of practices such as Baduanjin, Tai Chi, Yijinjing, and yoga, had the highest probability of improving LSBMD in postmenopausal women. These findings align with previous research by Li et al. (2023). Prior research reported that MBTs like Baduanjin may enhance musculoskeletal strength and flexibility, systematically engage joints and muscles, and improve balance, cardiopulmonary function, and mental wellbeing, thereby achieving holistic mind–body benefits (Liu et al., 2015). Some researchers hypothesize that these exercises could modulate endocrine function—for example, by elevating serum vitamin D levels, reducing parathyroid hormone, and increasing intestinal calcium absorption—mechanisms that could facilitate bone formation (Zhao et al., 2017). However, these mechanisms were not directly assessed in the present NMA and should therefore be interpreted as potential explanations rather than empirical evidence. Tai Chi, characterized by slow and fluid movements, has been reported to help prevent bone loss at the lumbar spine and proximal femoral neck in menopausal women (Zou et al., 2017). This benefit may be related to two mechanisms: first, engagement of the lumbar spine creates shear stress; second, repeated weight transfer between legs produces ground reaction forces (Wu and Hitt, 2005; Chang et al., 2014). Both mechanisms may stimulate osteogenesis in weight-bearing bones. Similarly, Yi Jin Jing, through stretching muscles and tendons, may enhance flexibility and joint mobility, potentially offering therapeutic benefits for bone health and overall wellbeing. Zou et al. (2017) noted that the unique low-impact nature of MBTs, such as Tai Chi and Baduanjin, not only supports bone metabolism and increases BMD but also minimizes injury risk, making them particularly suitable for elderly postmenopausal women (Shen et al., 2010). Considering physical, psychological, and social factors, MBTs such as Baduanjin and Tai Chi appear to be potentially valuable strategies for improving LSBMD in this demographic.

##### 4.1.1.2 FNBMD

This study indicated that MCT appears to be a potentially effective option for enhancing FNBMD in elderly postmenopausal women. MCT typically incorporates a combination of RT, CET, balance training, and flexibility exercises. Each component contributes independently, yet they act synergistically to improve both BMD and overall health. RT delivers mechanical loading that stimulates bone formation. CET enhances cardiovascular function and metabolic health. Balance training reduces the risk of falls, and flexibility exercises improve joint mobility and muscle coordination. Together, these components interact to facilitate gains in bone mineral density and overall physical wellbeing. Beyond these direct effects, potential biological mechanisms underlying the benefits of MCT warrant further attention. Previous studies suggest that comprehensive exercise programs can enhance blood circulation to bone tissue, thereby improving nutrient and oxygen delivery and supporting osteoblast activity (Renno et al., 2007). In addition, vascular endothelial cells can release local regulators such as nitric oxide, interleukin-6, and endothelin, which stimulate osteoblast differentiation and inhibit osteoclast activity. Importantly, recent experimental evidence highlights that mixed aerobic and anaerobic training protocols may also elicit endocrine responses that favor bone remodeling. For instance, Vasto et al. reported that high-intensity trampoline-based training increased circulating GLP-1 and GIP levels in adult women. These peptides are known to influence glucose metabolism and bone turnover (Vasto et al., 2022). Although such mechanisms were not directly assessed in our NMA, they provide valuable insights suggesting that the effects of MCT on bone health may extend beyond mechanical loading to include hormonal and metabolic pathways.

Nevertheless, these mechanisms remain speculative in the context of this analysis. Future RCTs incorporating both BMD outcomes and biomarker assessments are essential to elucidate whether these endocrine pathways mediate the observed benefits of MCT on skeletal health in postmenopausal women.

#### 4.1.2 Effects of different exercise interventions on secondary outcomes

SUCRA ranking identified CT and MBT as the most promising interventions for slowing the decline in WBBMD and THBMD, or the reduction of bone mass in postmenopausal women. A combination of CET and RT stimulates osteoblast activity through mechanical loading, promoting bone formation. Additionally, CET improves blood circulation and metabolism, and provides enhanced nourishment to the bones, collectively slowing the loss of WBBMD in postmenopausal women. MBTs, such as Baduanjin, Yijinjing, yoga, and Tai Chi, offer gentle mechanical loading to the bones, activating osteoblast activity and promoting bone formation. Furthermore, these exercises focus on balance, coordination, and muscle control, so that they are conducive to enhancing body stability and muscle strength, alleviating the risk of falls, and indirectly protecting bone health. Moreover, MBTs help alleviate stress, improve endocrine balance, and further attenuate the loss of THBMD in elderly postmenopausal women.

### 4.2 Differences in interventions and participants and their impact

Although exercise modalities were categorized as CET, RT, MCT, MBT, WBV, INT, and CT, the specific intervention protocols across studies varied in intensity, frequency, duration, loading pattern, and supervision (Supplementary Table S1). This heterogeneity may, in part, explain the differences in effect sizes. For example, WBV varied significantly in frequency and acceleration, potentially leading to inconsistent skeletal responses. Mind-body training, while generally producing lower impact loads, may improve lumbar spine stability and reduce fall risk, explaining its relative advantage in improving LSBMD rather than the hip. Intervention duration and adherence also influenced the results. Hence, these differences should be considered when interpreting the pooled results. More standardized reporting and protocol design is needed in future trials.

Collectively, the subgroup and sensitivity analyses indicate that participant characteristics have limited influence on the overall conclusions. Specifically, CET and WBV appear particularly beneficial in improving LSBMD among osteoporotic women, while MCT demonstrates relative advantages in enhancing FNBMD, especially in healthy participants. These findings are consistent with the exercise characteristics of osteoporotic populations: given their reduced bone mass and fragile skeletal architecture, osteoporotic women are typically unsuitable for high-impact or high-load exercise. Instead, moderate-intensity, continuous aerobic exercise (e.g., CET) offers a safe option that stimulates bone metabolism and slows bone loss (Papaioannou et al., 2010; Yagami et al., 2014). Moreover, this population often experiences decreased muscle strength and balance, leading to elevated fall risk. WBV provides skeletal loading under low mechanical stress and also improves lower-limb strength and balance, thereby enhancing bone density and reducing fall risk simultaneously (Rayman et al., 2011; Triunfo et al., 2013; Author anonymous, 2023). In summary, these results underscore the differential effects of exercise interventions across populations and highlight the need for future studies to design exercise prescriptions for osteoporotic women that are both safer and more effective.

## 5 Strengths and limitations

To the best of our knowledge, this NMA marks a pioneering endeavor to comprehensively evaluate and compare the diverse impacts of exercise interventions on BMD in elderly postmenopausal women, while simultaneously establishing a comprehensive ranking of these interventions based on their efficacy. This study unveils profound insights into the optimal interventions for fortifying BMD in this demographic. However, several limitations must be acknowledged. First, the relatively constrained sample sizes and the paucity of included studies could potentially compromise the precision and generalizability of the findings. Second, the considerable variability in exercise protocols, participant profiles, as well as the intensity, frequency, and duration of physical activity across studies introduces an element of heterogeneity. Further stratification by baseline BMD status or detailed age categories was not feasible due to insufficient data. Nevertheless, differences in baseline BMD levels and age distributions across studies may have influenced the observed outcomes. However, the limited reporting prevented more comprehensive exploration. In addition, the exclusive reliance on English-language literature may introduce potential selection bias. Therefore, more high-quality RCTs are necessary to validate the results of this study.

## 6 Conclusion

Currently, no single exercise intervention has been shown to unequivocally optimize BMD across all skeletal sites in elderly postmenopausal women. MBT emerged as a potentially effective intervention for slowing BMD decline and preserving bone mass in the lumbar spine and total hip joint. MCT appeared most promising for enhancing FNBMD, while CT showed potential benefits in sustaining WBBMD in postmenopausal women. Given the inherent constraints of current clinical research, future research should focus on larger cohorts, extended follow-up durations, and more stringent research frameworks to substantiate these promising yet preliminary findings.

## Data Availability

The original contributions presented in the study are included in the article/Supplementary Material, further inquiries can be directed to the corresponding author.
