# Supplementary material for: Effects of different exercise interventions on bone mineral density in elderly postmenopausal women: a network meta-analysis
Source: Front Physiol. 2025 Sep 25;16:1633913. doi: 10.3389/fphys.2025.1633913 (PMC12507884; doi:10.3389/fphys.2025.1633913)
Supplement: Supplementary file 1 [file Supplementaryfile1.docx]

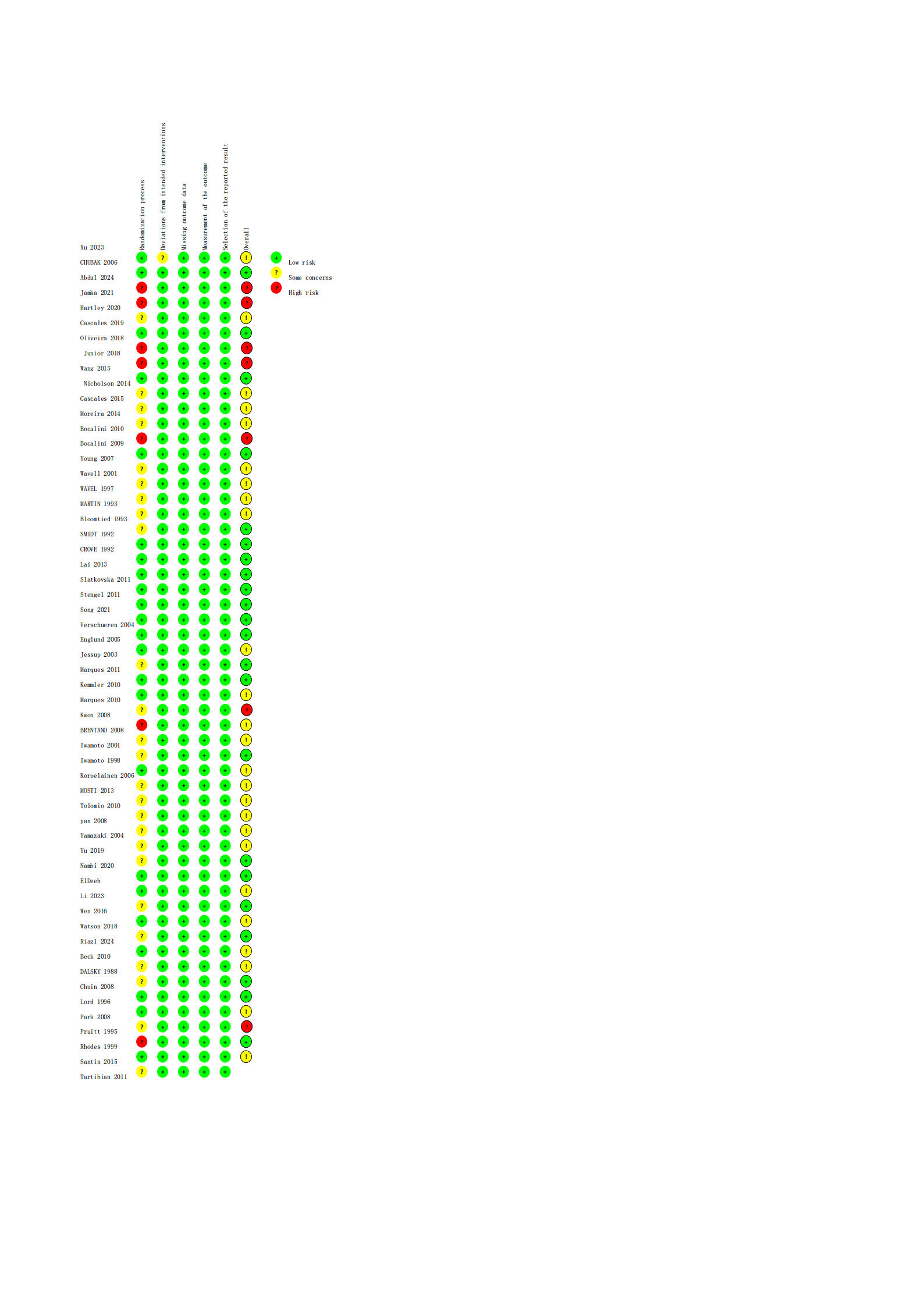


Figure S1 Risk of bias rating


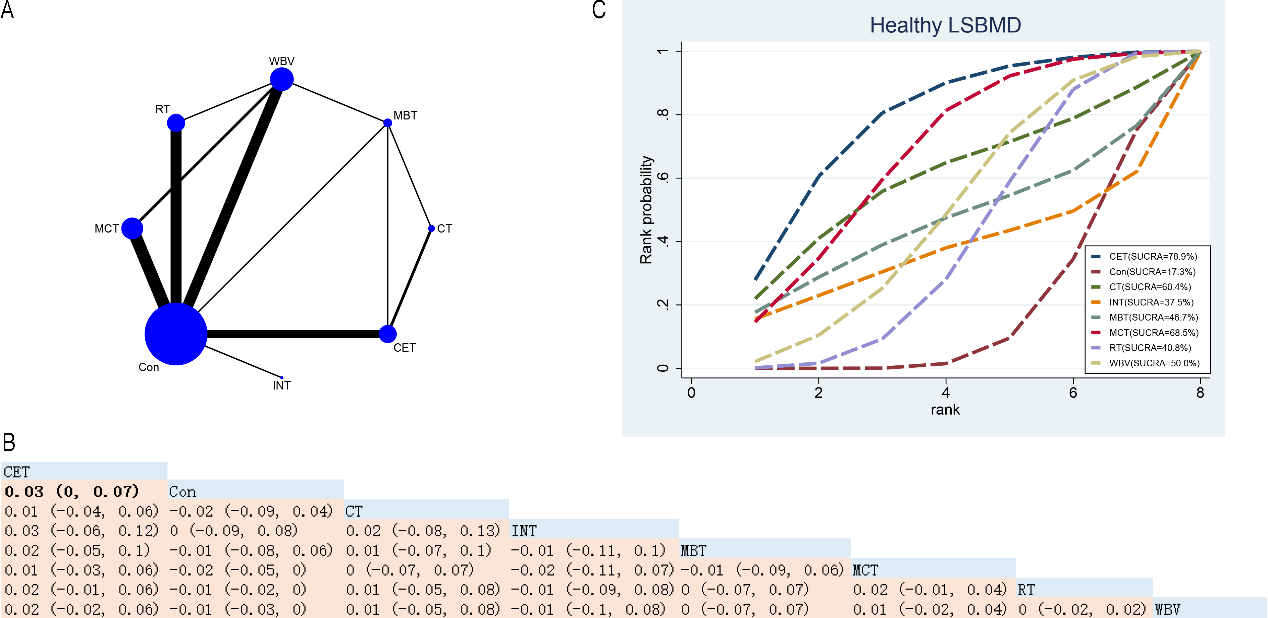


Figure S2 Network graph (A), league table (B), and probability plot (C) of LSBMD in the healthy population subgroup analysis


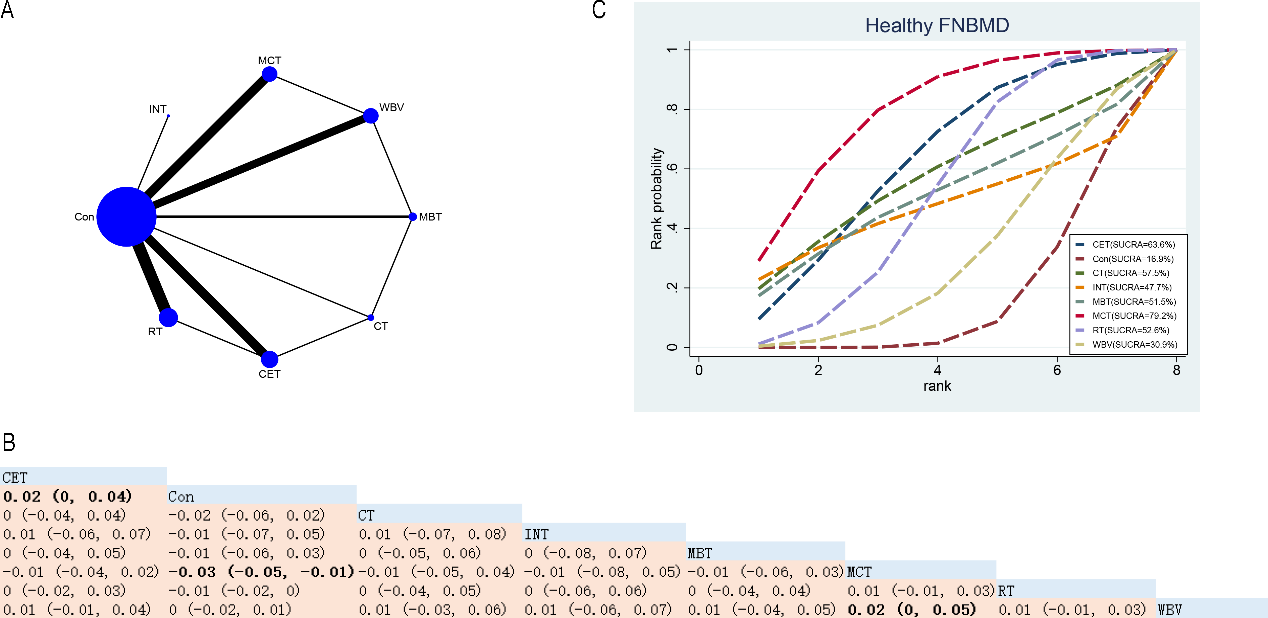


Figure S3 Network graph (A), league table (B), and probability plot (C) of FNBMD in the healthy population subgroup analysis


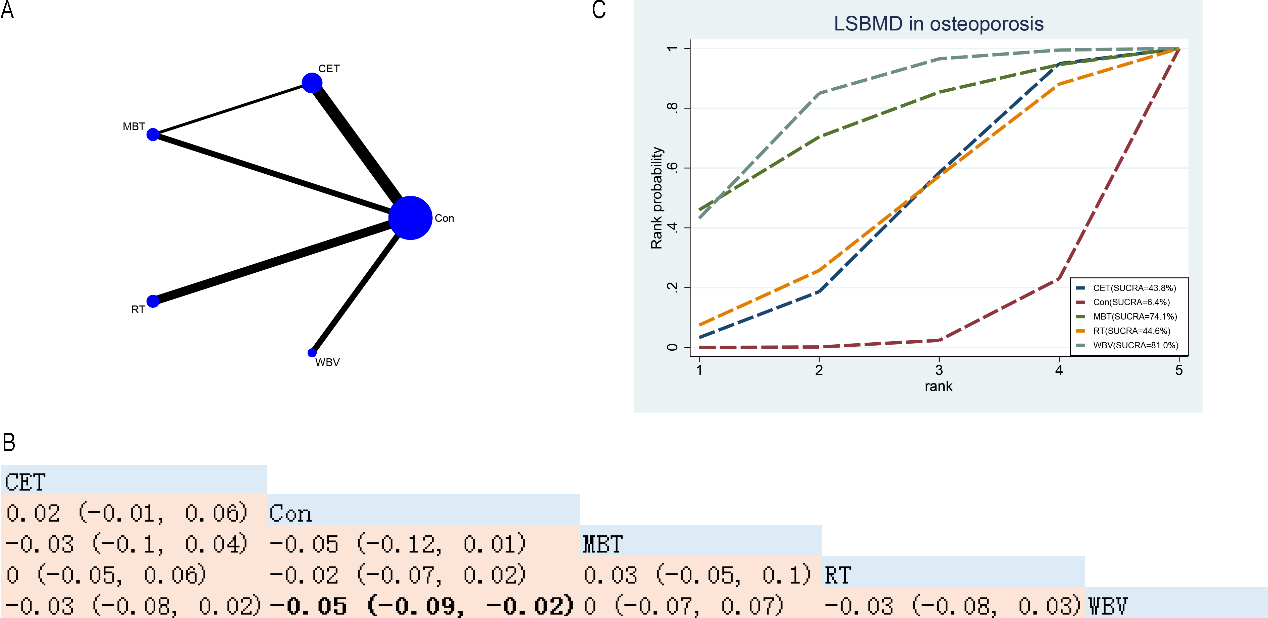


Figure S4 Network graph (A), league table (B), and probability plot (C) of LSBMD in the osteoporosis population subgroup analysis


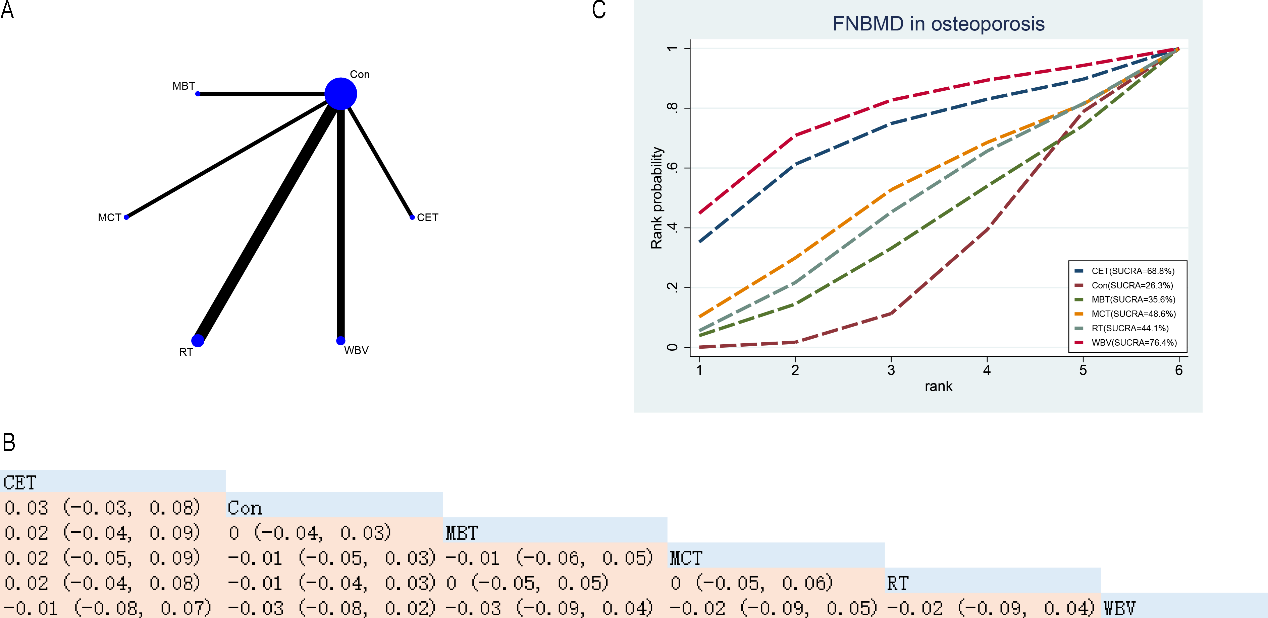


Figure S5 Network graph (A), league table (B), and probability plot (C) of FNBMD in the osteoporosis population subgroup analysis


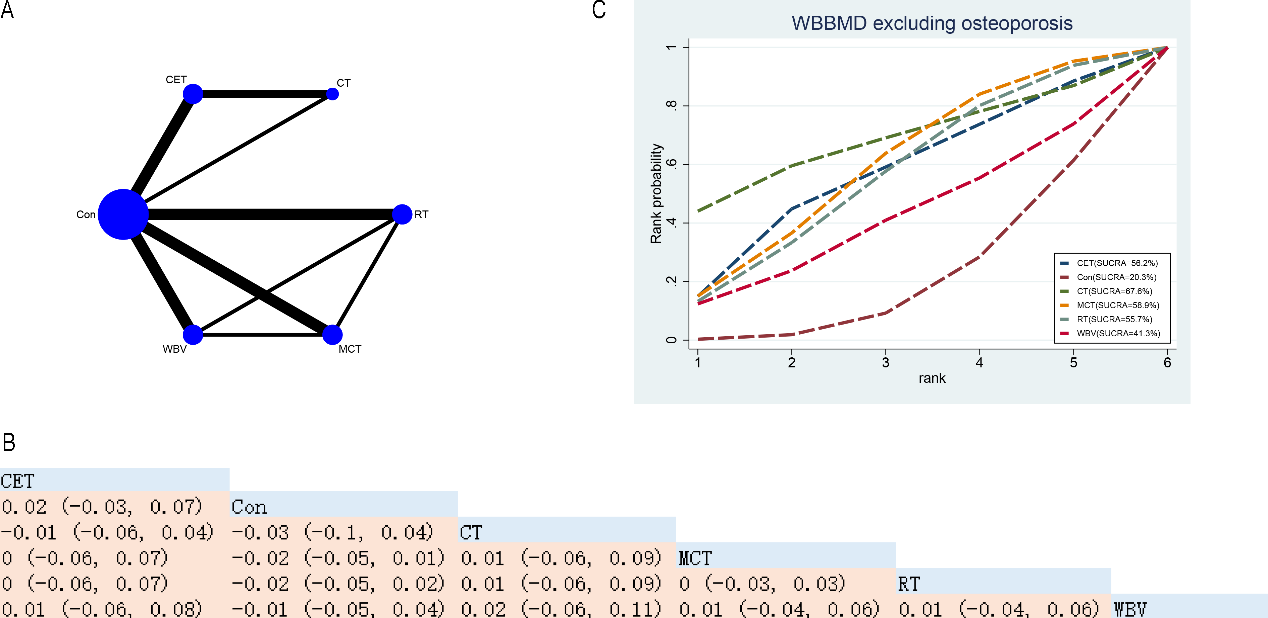


Figure S6 Network graph (A), league table (B), and probability plot (C) of WBBMD after excluding the osteoporosis population in the sensitivity analysis


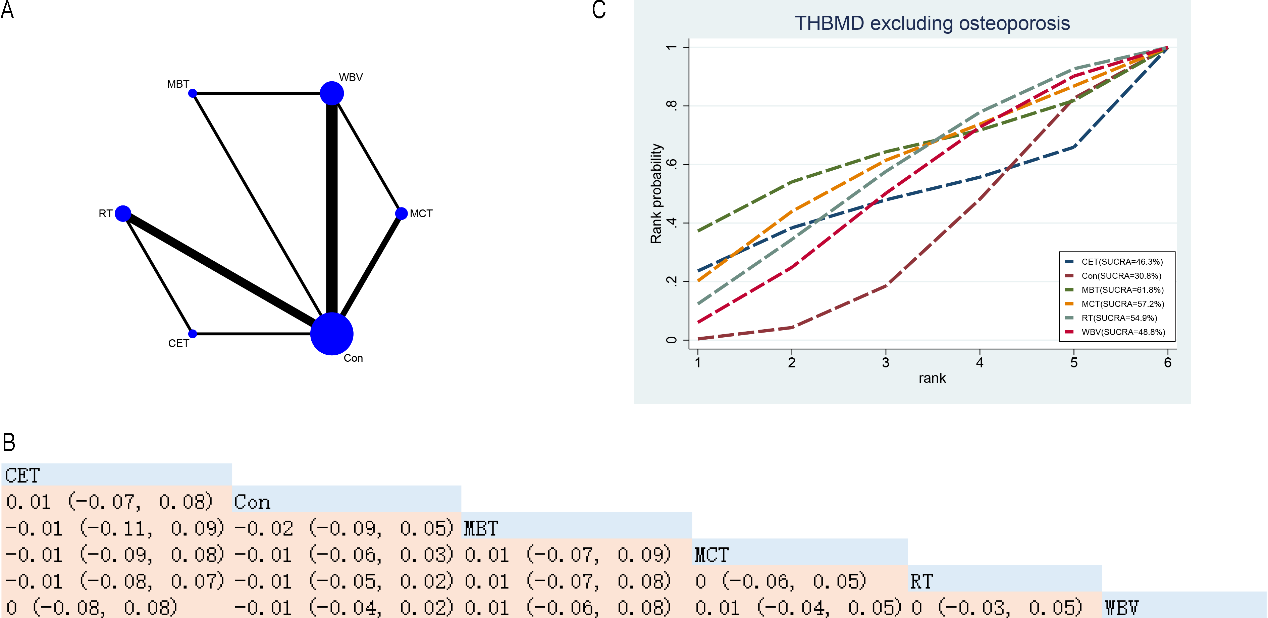


Figure S7 Network graph (A), league table (B), and probability plot (C) of THBMD after excluding the osteoporosis population in the sensitivity analysis.


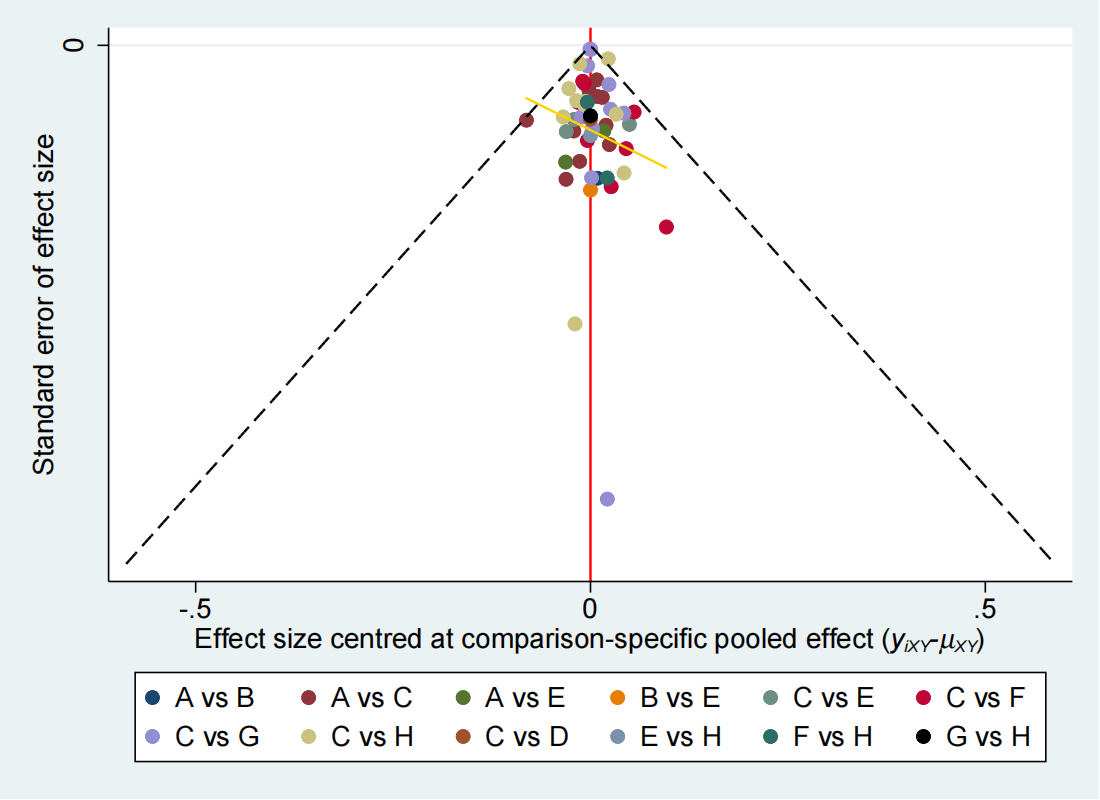


Figure S8 Comparatively adjusted funnel plot


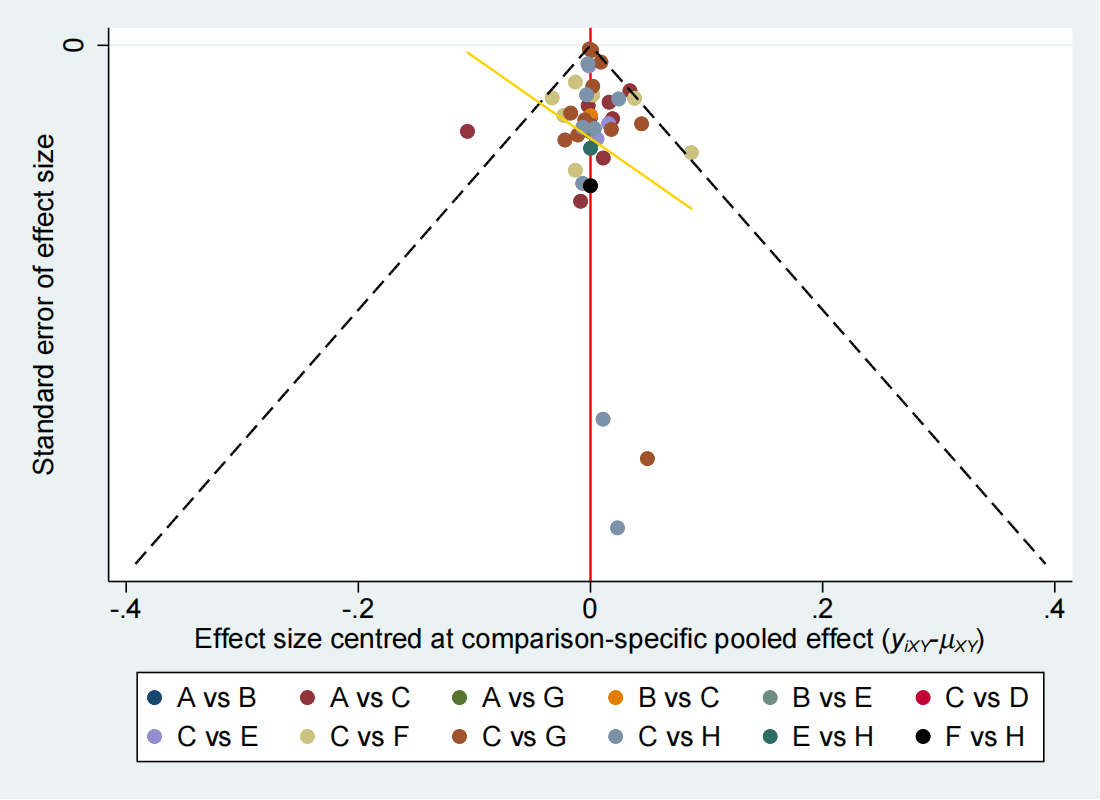


Figure S9 Comparatively adjusted funnel plot


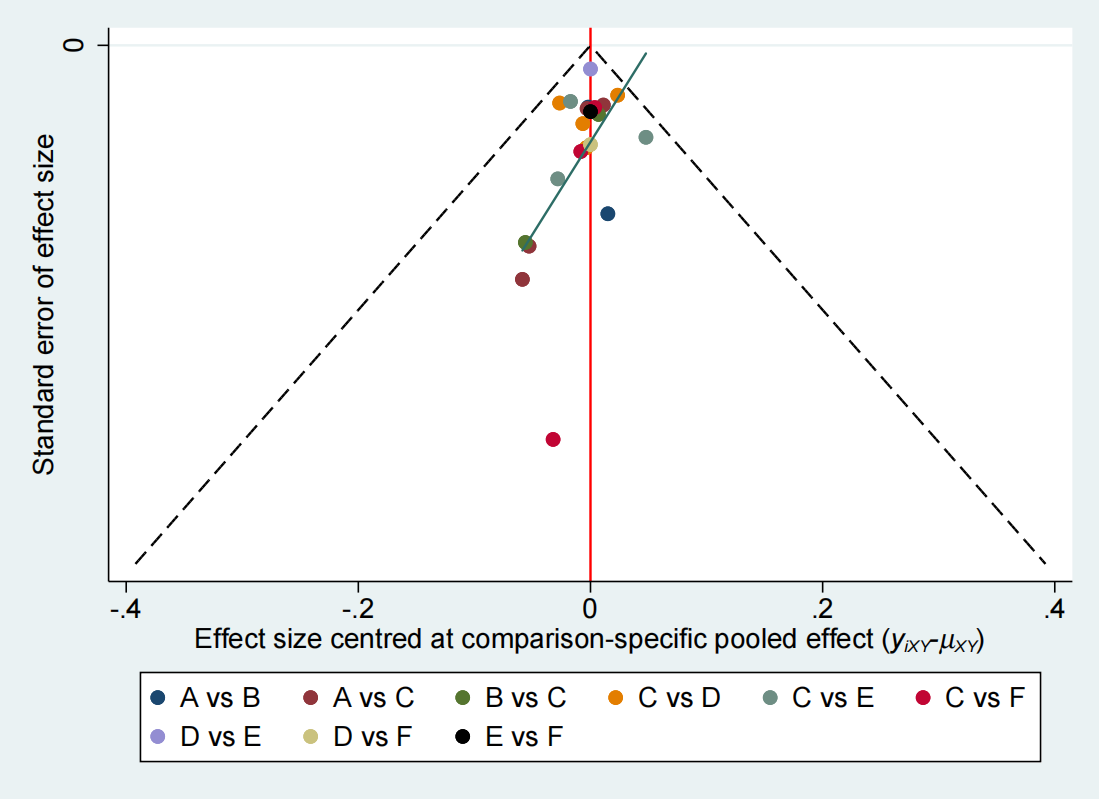


Figure S10 Comparatively adjusted funnel plot


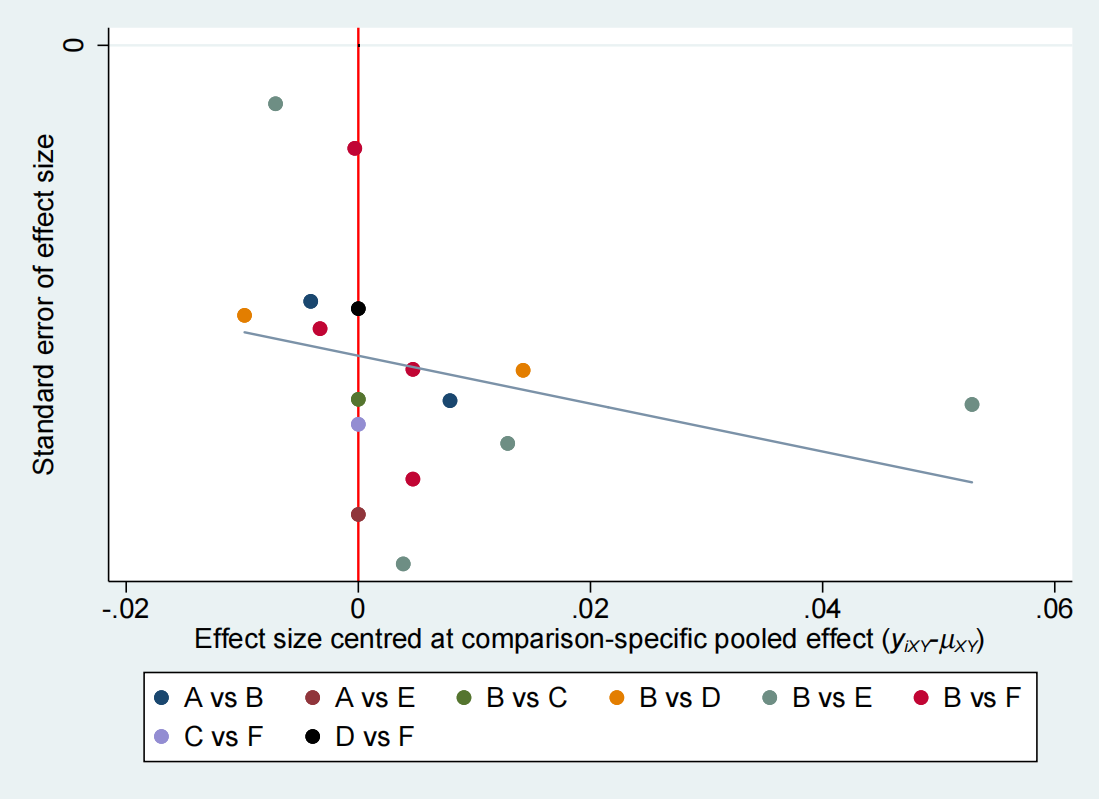


Figure S11 Comparatively adjusted funnel plot


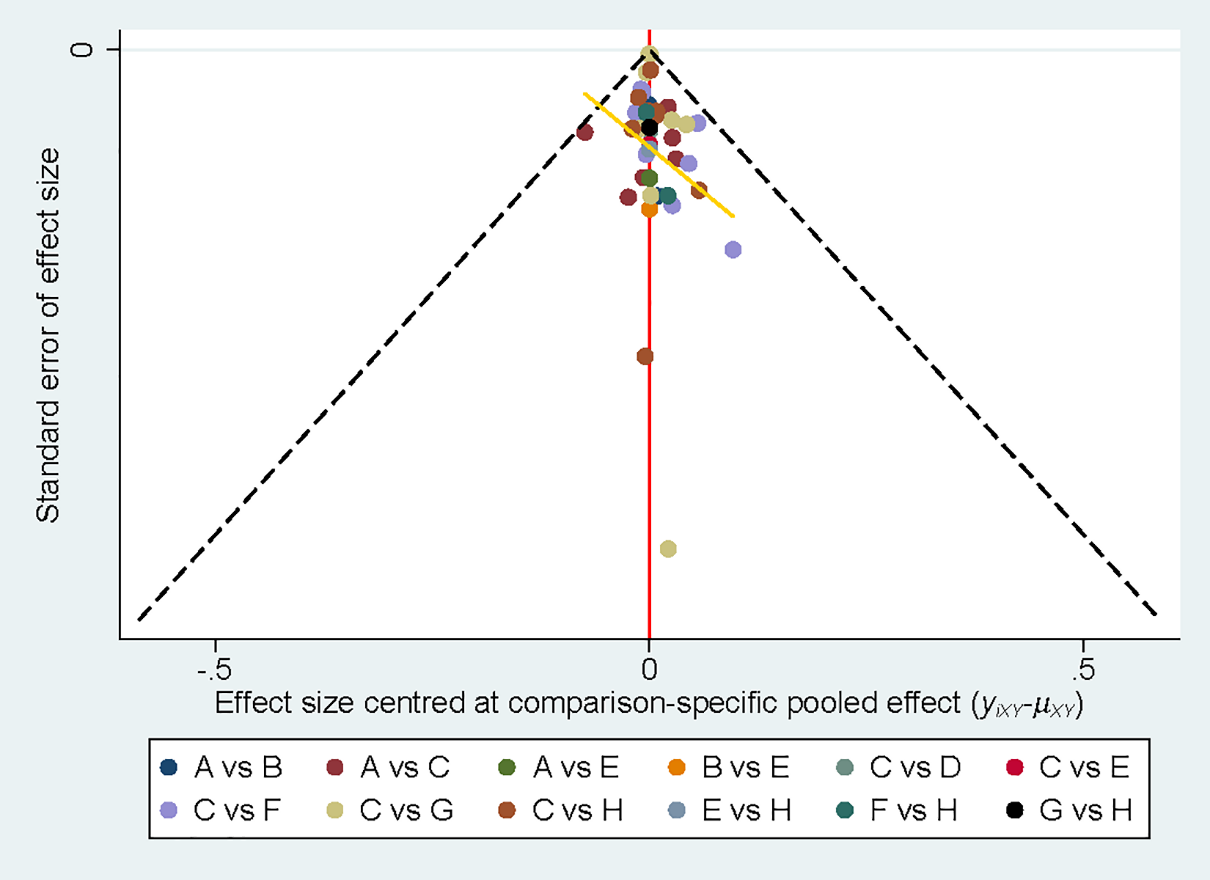


Figure S12 Funnel plot of LSBMD in the healthy population subgroup analysis.


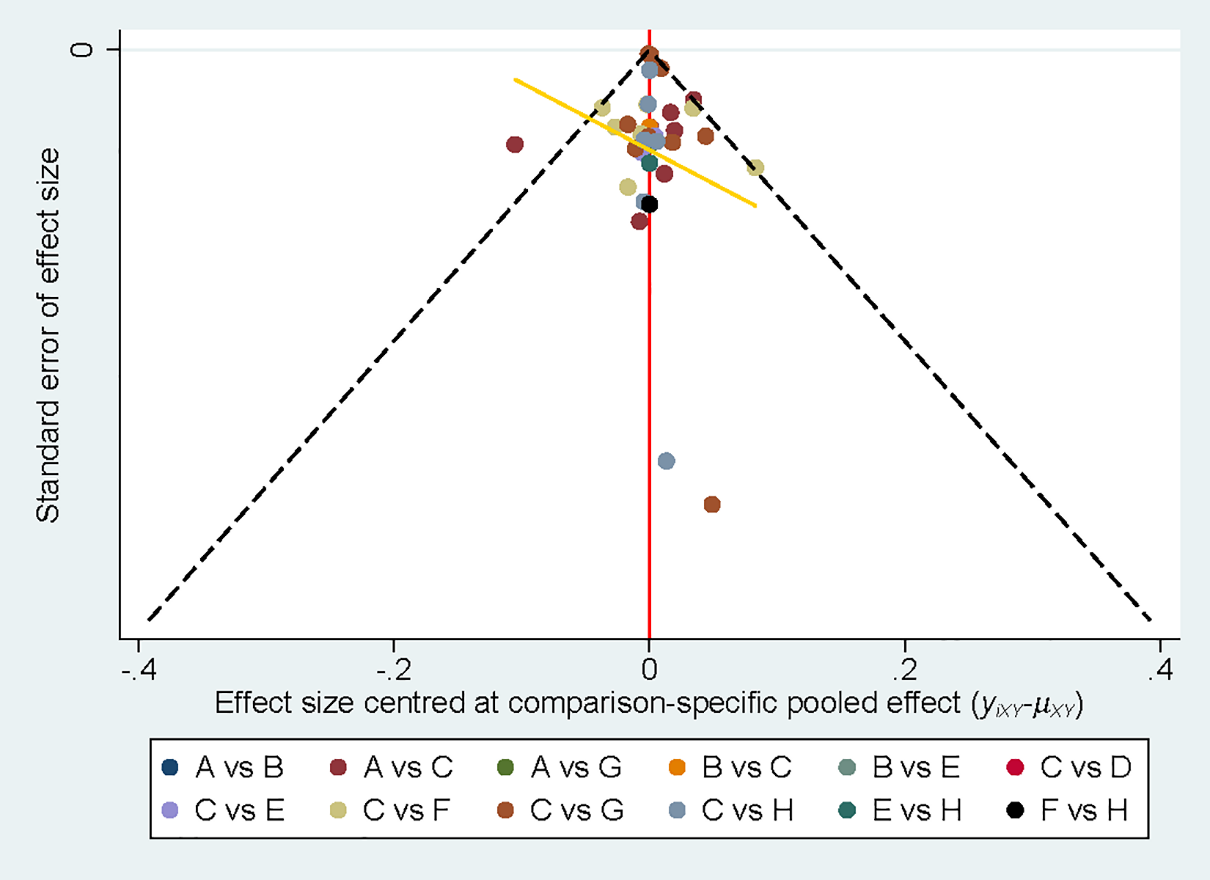


Figure S13 Funnel plot of FNBMD in the healthy population subgroup analysis.


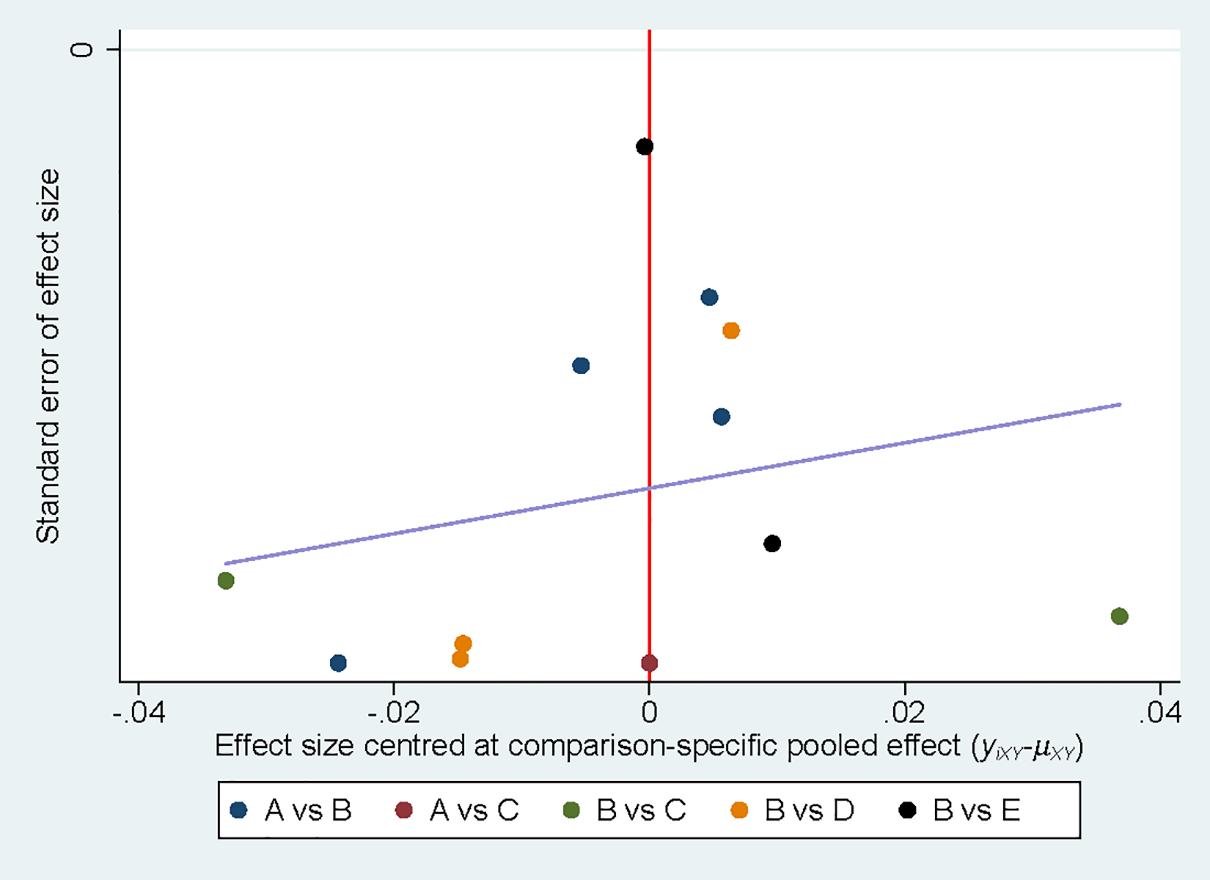


Figure S14 Funnel plot of LSBMD in the osteoporosis population subgroup analysis.


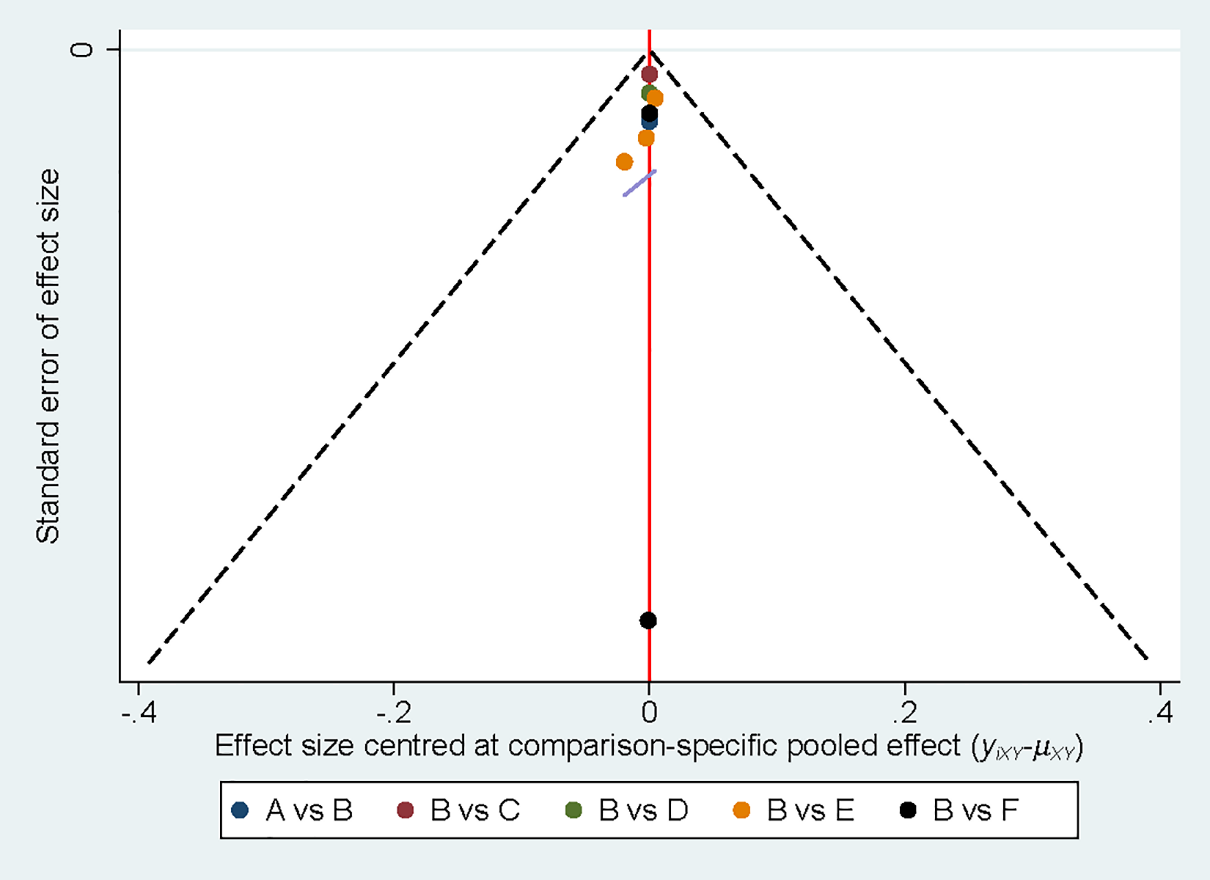


Figure S15 Funnel plot of FNBMD in the osteoporosis population subgroup analysis.


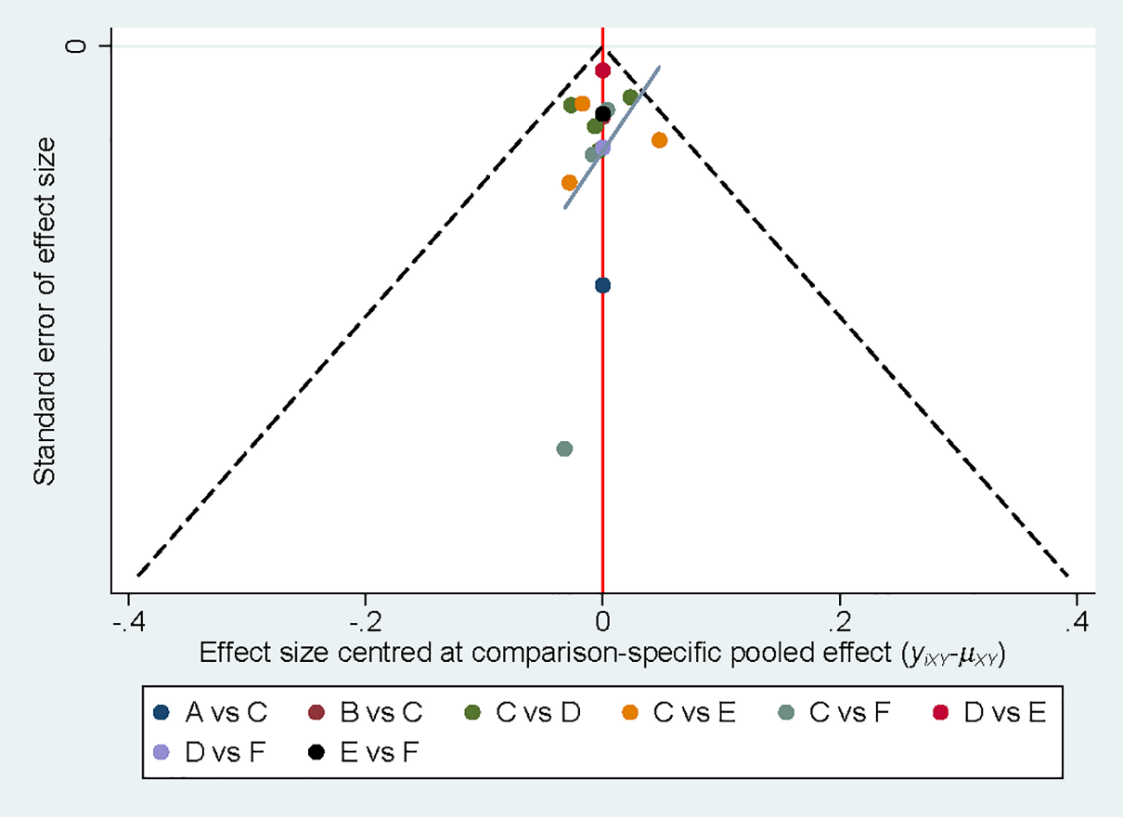


Figure S16 Funnel plot of WBBMD after excluding the osteoporosis population in the sensitivity analysis.


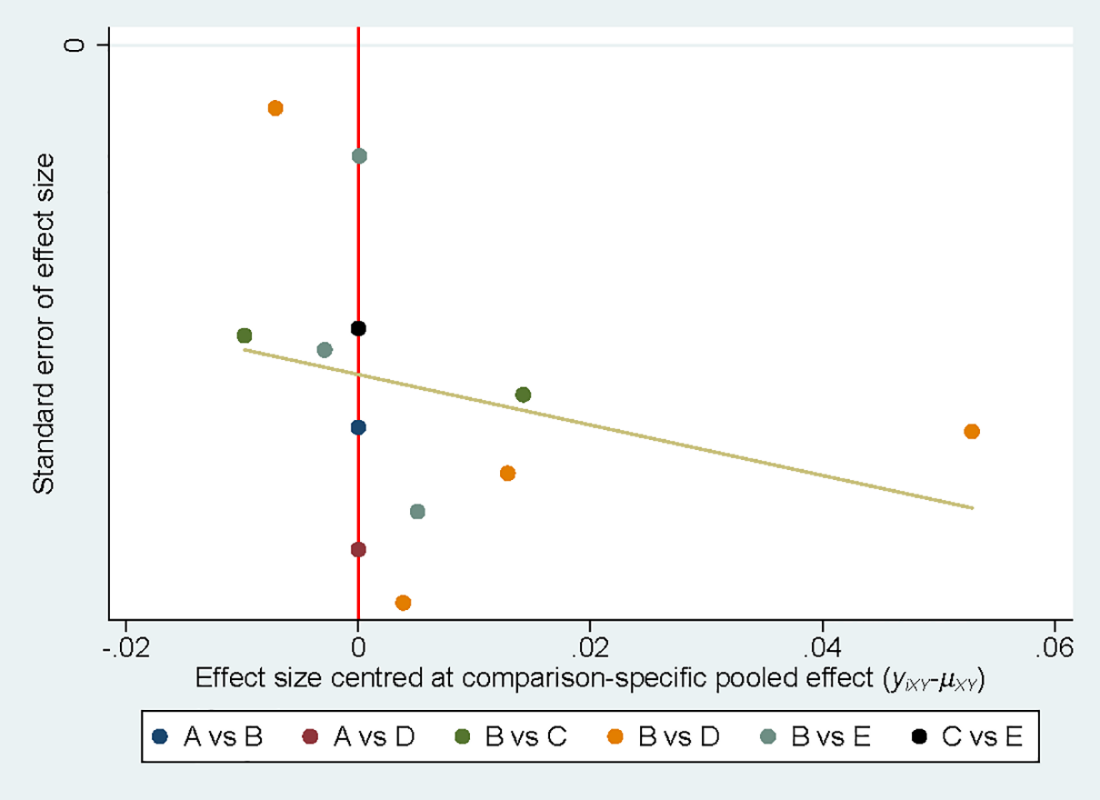


Figure S17 Funnel plot of THBMD after excluding the osteoporosis population in the sensitivity analysis.

**Appendix A: Search strategies**

**Pubmed:2204 records**

**#1**"Postmenopause"[Mesh]

**#2**"Post-Menopause*"[Title/Abstract] OR "Postmenopause"[Title/Abstract] OR "postmenopausal women"[Title/Abstract] OR "Post-menopausal Period"[Title/Abstract] OR "Postmenopausal Period"[Title/Abstract] OR "postmenopausal female"[Title/Abstract] OR "post menopause"[Title/Abstract] OR "Post menopausal Period"[Title/Abstract]

**#3** #1 OR #2

**#4**"Exercise"[Mesh]

**#5**"Physical Exercise"[Title/Abstract] OR "physical work-out"[Title/Abstract] OR "physical workout"[Title/Abstract] OR "physical exertion"[Title/Abstract] OR "physical exercise*"[Title/Abstract] OR "physical effort"[Title/Abstract] OR "Physical Activit*"[Title/Abstract] OR "Isometric Exercise*"[Title/Abstract] OR "fitness workout"[Title/Abstract] OR "fitness training"[Title/Abstract] OR "exertion"[Title/Abstract] OR "Exercise*"[Title/Abstract] OR "Exercise Training*"[Title/Abstract] OR "exercise performance"[Title/Abstract] OR "exercise capacity"[Title/Abstract] OR "effort"[Title/Abstract] OR "biometric exercise"[Title/Abstract] OR "Aerobic Exercise*"[Title/Abstract] OR "Acute Exercise*"[Title/Abstract]

**#6** #4 OR #5

**#7**"Resistance Training"[Mesh]

**#8** "Weight Lifting Strengthening Program"[Title/Abstract] OR "Weight Lifting Exercise Program"[Title/Abstract] OR "Weight Bearing Strengthening Program"[Title/Abstract] OR "Weight-Bearing Exercise Program"[Title/Abstract] OR "strength-type training"[Title/Abstract] OR "strength-type exercise"[Title/Abstract] OR "Strength Training"[Title/Abstract] OR "resistance-type training"[Title/Abstract] OR "resistance-type exercise"[Title/Abstract] OR "Resistance Training"[Title/Abstract] OR "resistance exercise training"[Title/Abstract] OR "resistance exercise"[Title/Abstract]

**#9** #7 OR #8

**#10** "Muscle Strength"[Mesh]

**#11** "muscular strength"[Title/Abstract] OR "muscular power"[Title/Abstract] OR "muscular force"[Title/Abstract] OR "muscular dynamic strength"[Title/Abstract] OR "Muscle Strength"[Title/Abstract] OR "muscle power"[Title/Abstract] OR "muscle force velocity relationship"[Title/Abstract] OR "muscle force"[Title/Abstract] OR "muscle dynamic strength"[Title/Abstract] OR "Arthrogenic Muscle Inhibition*"[Title/Abstract]

**#12** #10 OR #11

**#13** "Circuit-Based Exercise"[Mesh]

**#14** "circuit-type training"[Title/Abstract] OR "circuit-type exercise"[Title/Abstract] OR "circuit-based training"[Title/Abstract] OR "Circuit Training"[Title/Abstract] OR "Circuit Based Exercise"[Title/Abstract]

**#15** #13 OR #14

**#16** "step aerobics"[Title/Abstract] OR "low impact aerobics"[Title/Abstract] OR "low impact aerobic exercise"[Title/Abstract] OR "aerobics exercise"[Title/Abstract] OR "aerobics"[Title/Abstract] OR "aerobic exercise"[Title/Abstract] OR "aerobic dance*"[Title/Abstract]

**#17** "anaerobic exercise"[Title/Abstract] OR "anaerobic exercise work"[Title/Abstract] OR "anaerobic work"[Title/Abstract]

**#18** "physical activity"[Title/Abstract]

**#19** #6 OR #9 OR #12 OR #15 OR #16 OR #17 OR #18

**#20** "Random*"[Title/Abstract]

**#21** "RCT"[Title/Abstract]

**#22** "Randomized control trial"[Title/Abstract]

**#23** "Control*"[Title/Abstract]

**#24** #20 OR #21 OR #22 OR #23

**#25** #3 AND #19 AND #24

**Cochrane Library: 2895 records**

**#1** MeSH descriptor: [Exercise] explode all trees

**#2** (('Physical Exercise' OR 'physical work-out' OR 'physical workout' OR 'physical exertion' OR 'physical exercise*' OR 'physical effort' OR 'Physical Activit*' OR 'Isometric Exercise*' OR 'fitness workout' OR 'fitness training' OR 'exertion' OR 'Exercise*' OR 'Exercise Training*' OR 'exercise performance' OR 'exercise capacity' OR 'effort' OR 'biometric exercise' OR 'Aerobic Exercise*' OR 'Acute Exercise*' )):ti,ab,kw

**#3** #1 OR #2

**#4** MeSH descriptor: [Resistance Training] explode all trees

**#5** (('Weight Lifting Strengthening Program' OR 'Weight Lifting Exercise Program' OR 'Weight Bearing Strengthening Program' OR 'Weight-Bearing Exercise Program' OR 'strength-type training' OR 'strength-type exercise' OR 'Strength Training' OR 'resistance-type training' OR 'resistance-type exercise' OR 'Resistance Training' OR 'resistance exercise training' OR 'resistance exercise' )):ti,ab,kw

**#6** #4 OR #5

**#7** MeSH descriptor: [Muscle Strength] explode all trees

**#8** (('muscular strength' OR 'muscular power' OR 'muscular force' OR 'muscular dynamic strength' OR 'Muscle Strength' OR 'muscle power' OR 'muscle force velocity relationship' OR 'muscle force' OR 'muscle dynamic strength' OR 'Arthrogenic Muscle Inhibition*')):ti,ab,kw

**#9** #7 OR #8

**#10** MeSH descriptor: [Circuit-Based Exercise] explode all trees

**#11** (('circuit-type training' OR 'circuit-type exercise' OR 'circuit-based training' OR 'Circuit Training' OR 'Circuit Based Exercise')):ti,ab,kw

**#12** #10 OR #11

**#13** (('physical activity')):ti,ab,kw

**#14** (('step aerobics' OR 'low impact aerobics' OR 'low impact aerobic exercise' OR 'aerobics exercise' OR 'aerobics' OR 'aerobic exercise' OR 'aerobic dance*')):ti,ab,kw

**#15** (('anaerobic exercise' OR 'anaerobic exercise work' OR 'anaerobic work')):ti,ab,kw

**#16** #13 OR #14 OR #15

**#17** #3 OR #6 OR #9 OR #12 OR #16

**#18** MeSH descriptor: [Postmenopause] explode all trees

**#19** (('Post-Menopause*' OR 'Postmenopause' OR 'postmenopausal women' OR 'Post-menopausal Period' OR 'Postmenopausal Period' OR 'postmenopausal female' OR 'post menopause' OR 'Post menopausal Period')):ti,ab,kw

**#20** #18 OR #19

**#21** (('Random*')):ti,ab,kw

**#22** (('RCT')):ti,ab,kw

**#23** (('Randomized control trial')):ti,ab,kw

**#24** (('Control*')):ti,ab,kw

**#25** #21 OR #22 OR #23 OR #24

**#26** #17 AND #20 AND #25

**Web of Science: 4431 records**

**#1** TS=((Physical Exercise) OR (physical work-out) OR (physical workout) OR (physical exertion) OR (physical exercise*) OR (physical effort) OR (Physical Activit*) OR (Isometric Exercise*) OR (fitness workout) OR (fitness training) OR (exertion) OR (Exercise*) OR (Exercise Training*) OR (exercise performance) OR (exercise capacity) OR (effort) OR (biometric exercise) OR (Aerobic Exercise*) OR (Acute Exercise* )) and Preprint Citation Index (Exclude – Database)

**#2** TS=((Post-Menopause*) OR (Postmenopause) OR (postmenopausal women) OR (Post-menopausal Period) OR (Postmenopausal Period) OR (postmenopausal female) OR (post menopause) OR (Post menopausal Period)) and Preprint Citation Index (Exclude – Database)

**#3** TS=((Weight Lifting Strengthening Program) OR (Weight Lifting Exercise Program) OR (Weight Bearing Strengthening Program) OR (Weight-Bearing Exercise Program) OR (strength-type training) OR (strength-type exercise) OR (Strength Training) OR (resistance-type training) OR (resistance-type exercise) OR (Resistance Training) OR (resistance exercise training) OR (resistance exercise)) and Preprint Citation Index (Exclude – Database)

**#4** TS=((muscular strength) OR (muscular power) OR (muscular force) OR (muscular dynamic strength) OR (Muscle Strength) OR (muscle power) OR (muscle force velocity relationship) OR (muscle force) OR (muscle dynamic strength) OR (Arthrogenic Muscle Inhibition*)) and Preprint Citation Index (Exclude – Database)

**#5** TS=((circuit-type training) OR (circuit-type exercise) OR (circuit-based training) OR (Circuit Training) OR (Circuit Based Exercise)) and Preprint Citation Index (Exclude – Database)

**#6** TS=((step aerobics) OR (low impact aerobics) OR (low impact aerobic exercise) OR (aerobics exercise) OR (aerobics) OR (aerobic exercise) OR (aerobic dance*)) and Preprint Citation Index (Exclude – Database)

**#7** TS=(('physical activity')) and Preprint Citation Index (Exclude – Database)

**#8** TS=((anaerobic exercise) OR (anaerobic exercise work) OR (anaerobic work)) and Preprint Citation Index (Exclude – Database)

**#9** #1 OR #3 OR #4 OR #5 OR #6 OR #7 OR #8 and Preprint Citation Index (Exclude – Database)

**#10** TS=((Random*)) and Preprint Citation Index (Exclude – Database)

**#11** TS=((RCT)) and Preprint Citation Index (Exclude – Database)

**#12** TS=((Randomized control trial)) and Preprint Citation Index (Exclude – Database)

**#13** TS=((Control*)) and Preprint Citation Index (Exclude – Database)

**#14** #10 OR #11 OR #12 OR #13 and Preprint Citation Index (Exclude – Database)

**#15** #2 AND #9 AND #14 and Preprint Citation Index (Exclude – Database)

**EMBASE:3760 records**

**#1** 'exercise'/exp

**#2** 'physical exercise':ti,ab,kw OR 'physical work-out':ti,ab,kw OR 'physical workout':ti,ab,kw OR 'physical exertion':ti,ab,kw OR 'physical exercise*':ti,ab,kw OR 'physical effort':ti,ab,kw OR 'physical activit*':ti,ab,kw OR 'isometric exercise*':ti,ab,kw OR 'fitness workout':ti,ab,kw OR 'fitness training':ti,ab,kw OR 'exertion':ti,ab,kw OR 'exercise*':ti,ab,kw OR 'exercise training*':ti,ab,kw OR 'exercise performance':ti,ab,kw OR 'exercise capacity':ti,ab,kw OR 'effort':ti,ab,kw OR 'biometric exercise':ti,ab,kw OR 'aerobic exercise*':ti,ab,kw OR 'acute exercise*':ti,ab,kw

**#3** #1 OR #2

**#4** 'resistance training'/exp

**#5** 'weight lifting strengthening program':ti,ab,kw OR 'weight lifting exercise program':ti,ab,kw OR 'weight bearing strengthening program':ti,ab,kw OR 'weight-bearing exercise program':ti,ab,kw OR 'strength-type training':ti,ab,kw OR 'strength-type exercise':ti,ab,kw OR 'strength training':ti,ab,kw OR 'resistance-type training':ti,ab,kw OR 'resistance-type exercise':ti,ab,kw OR 'resistance training':ti,ab,kw OR 'resistance exercise training':ti,ab,kw OR 'resistance exercise':ti,ab,kw

**#6** #4 OR #5

**#7** 'muscle strength'/exp

**#8** 'muscular strength':ti,ab,kw OR 'muscular power':ti,ab,kw OR 'muscular force':ti,ab,kw OR 'muscular dynamic strength':ti,ab,kw OR 'muscle strength':ti,ab,kw OR 'muscle power':ti,ab,kw OR 'muscle force velocity relationship':ti,ab,kw OR 'muscle force':ti,ab,kw OR 'muscle dynamic strength':ti,ab,kw OR 'arthrogenic muscle inhibition*':ti,ab,kw

**#9** #7 OR #8

**#10** 'circuit training'/exp

**#11** 'circuit-type training':ti,ab,kw OR 'circuit-type exercise':ti,ab,kw OR 'circuit-based training':ti,ab,kw OR 'circuit training':ti,ab,kw OR 'circuit based exercise':ti,ab,kw

**#12** #10 OR #11

**#13** 'aerobic exercise'/exp

**#14** 'step aerobics':ti,ab,kw OR 'low impact aerobics':ti,ab,kw OR 'low impact aerobic exercise':ti,ab,kw OR 'aerobics exercise':ti,ab,kw OR 'aerobics':ti,ab,kw OR 'aerobic exercise':ti,ab,kw OR 'aerobic dance*':ti,ab,kw

**#15** #13 OR #14

**#16** 'anaerobic exercise'/exp

**#17** 'anaerobic exercise':ti,ab,kw OR 'anaerobic exercise work':ti,ab,kw OR 'anaerobic work':ti,ab,kw

**#18** #16 OR #17

**#19** 'physical activity'/exp

**#20** 'physical activity':ti,ab,kw

**#21** #19 OR #20

**#22** #3 OR #6 OR #9 OR #12 OR #15 OR #18 OR #21

**#23** 'postmenopause'/exp

**#24** 'post-menopause*':ti,ab,kw OR 'postmenopause':ti,ab,kw OR 'postmenopausal women':ti,ab,kw OR 'post-menopausal period':ti,ab,kw OR 'postmenopausal period':ti,ab,kw OR 'postmenopausal female':ti,ab,kw OR 'post menopause':ti,ab,kw OR 'post menopausal period':ti,ab,kw

**#25** #23 OR #24

**#26** 'random*':ti,ab,kw

**#27** 'rct':ti,ab,kw

**#28** 'randomized control trial':ti,ab,kw

**#29** 'control*':ti,ab,kw

**#30** #26 OR #27 OR #28 OR #29

**#31** #22 AND #25 AND #30

Table S1 Intervention details

| First authors | Years published | Country | Treatment-specific design (specific content and operation, frequency, etc.) | | Treatment time |
| --- | --- | --- | --- | --- | --- |
|  |  |  | Interventions | Check |  |
| Abdul | 2024 | Lebanon | Train twice a week with at least one day of rest between sessions for one year. Perform five repetitions at 85% of 1-RM with the concentric phase (lifting phase) at a self-selected speed. | Control group (without exercise) | I trained twice a week, with non-consecutive training days, for a year. |
| Bocalini | 2010 | Brazil | In the first week, all subjects in the resistance training group started with a load of 40% of their 1RM for each exercise. The load was gradually increased until the subjects could complete three sets of 10 to 12 repetitions at 60% to 70% of their 1RM for each exercise. | Control group (without exercise) | Three one-hour training sessions per week, with non-consecutive training days, for 24 weeks. |
| Bocalini | 2009 | Brazil | Each training session commenced with a 10-minute warm-up, followed by one set of exercises performed at 50% of the one-repetition maximum (1RM). The load was progressively increased until participants were able to complete three sets of 10 repetitions at 85% of their 1RM for the specified movement. | Control group (without exercise) | Resistance training was performed three times per week (each session lasting 1 hour) on non-consecutive days over a 24-week period. |
| Bloomfield | 1993 | USA | Each class includes 15 minutes of warm-up activities, which consist of flexibility and aerobics exercises, 30 minutes of stationary bike riding, and a five-minute walk for relaxation. | Control group (without exercise) | For eight consecutive months, three times a week, every other day. |
| BRENTANO | 2008 | Brazil | Each exercise is performed separately. Take a 2-minute break between sets. Repeat each set 20 to 6 times. The load should be 45% to 80% of 1RM. Do 2 to 4 sets for each exercise.  Perform the same exercise continuously without rest between sets. Repeat each set 20 to 10 times, with a load of 45 to 60% of 1RM. Do 2 to 3 sets for each exercise. | Control group (without exercise) | Train for 24 weeks, 3 times a week, 1 hour each time. |
| Beck | 2010 | Australia | The peak-to-peak acceleration at the set 30 Hz is 0.3g (the root mean square power average is 0.106 m/s²). | Control group (without exercise) | Receive supervised whole-body vibration (WBV) training twice a week for eight months. |
| CHUBAK | 2006 | USA | The exercise prescription consisted of at least 45 min of moderate-intensity exercise, 5dIwkj1 for 12 months. Participants were required to attend three supervised sessions per week at a study facility (University of Washington or a commercial gym) during months 1–3 and to exercise 2 dIwkj1 at home. For months 4–12, they were required to attend at least one of the three sessions offered weekly at a study facility and to exercise 4dIwkj1 either at home or at the facility. | Control participants attended 45-min stretching sessions once a week for the 12 months and were asked not to change other exercise habits. | For 12 consecutive months, three times a week at the gym and twice a week at home, with each session lasting at least 45 minutes. |
| Cascales | 2019 | Spain | On the sinusoidal vertical vibration platform (Power Plate), participants stood with their feet side by side, knees and hips flexed at 120°, arms crossed with shoulders flexed at 90°, and performed dynamic heel raises, with the rhythm controlled by a metronome. | The training content includes the monitoring of drop jump height (in centimeters), walking time (in minutes), and maximum heart rate reserve (%).  Control group (without exercise) | The training is conducted three times a week (non-consecutive days) for 24 weeks. The total number of training sessions is 72. |
| Cascales | 2015 | Spain | The parameters of amplitude (4 mm), working time (60 seconds) and recovery time (60 seconds) remained unchanged, with the training intensity set at 35 Hz. The first two weeks were the familiarization stage, with the working time being 45 seconds. | After squat jumps, aerobic exercise is performed. Over a period of 12 weeks, the load gradually increases. Participants walk at an intensity of 50% to 60% of their maximum heart rate minus their resting heart rate, with each exercise session lasting between 30 and 45 minutes.  Control group (without exercise) | The total duration of the intervention stage was 12 weeks. The experimental group received 3 training sessions per week, with a total of 36 training sessions. The rest time between each training day was at least 24 hours and at most 72 hours. |
| Chuin | 2009 | Canada | The training requirements are 60 minutes, including 15 minutes of warm-up and 45 minutes of resistance training. Each exercise consists of 3 sets of 8 repetitions, with an intensity of 80% of 1RM. Rest for 90 to 120 seconds between sets. Assess 1RM every 4 weeks to adjust the intensity. | Control group (without exercise) | The six-month resistance training program consists of three supervised training sessions per week, each lasting 60 minutes, with one day of rest between training days. |
| Dalsky | 1988 | USA | Weight-bearing exercise should last for 50 to 60 minutes. In the first three months, it includes walking, jogging, alternating walking and jogging, or treadmill walking, with an intensity of 60% to 70% of the maximum oxygen uptake. After three months, add stair climbing (8 repetitions, 88 steps each time, with jogging in between repetitions), and combine it with 15 to 20 minutes of non-weight-bearing activities, three times a week. | Control group (without exercise) | The program lasts for 9 months, and the participants attend three exercise classes each week. |
| ElDeeb | 2019 | Egypt | The vertical vibration frequency of the platform is 20 to 35 hertz, and the amplitude is 2.5 to 5 millimeters. | Control group (without exercise) | The whole-body vibration group received whole-body vibration training twice a week for 24 weeks. All participants were required to maintain their normal daily activities. |
| Englund | 2005 | Sweden | This program is accompanied by music and lasts for 50 minutes. It begins with a 10-minute warm-up, followed by a 27-minute combination of aerobic, strengthening, balance and coordination training, and concludes with an 11-minute relaxation, stretching and cool-down session. | Control group (without exercise) | It lasts for 12 months, with a 5-week break during the summer vacation. The project is carried out with musical accompaniment and lasts for 50 minutes. |
| GROVE | 1992 | USA | Each workout consists of 15 to 20 minutes of warm-up activities, 20 minutes of low-intensity exercise and 15 minutes of relaxation. | Each workout consists of 15 to 20 minutes of warm-up activities, 20 minutes of high-intensity exercise and 15 minutes of relaxation.  Control group (without exercise) | Participate in a supervised exercise program three times a week for about an hour each time for a year. |
| Hartley | 2020 | UK | Six months of unilateral high-intensity exercise, gradually increasing to 50 multi-directional jumps per day, each lasting 3-4 minutes, divided into 3-5 sets, with 15 seconds of rest between sets, and the total exercise time was less than 15 minutes. | Control group (without exercise) | 15 minutes every day for six months |
| Iwamoto | 2001 | Japan | The training requires women to measure their daily steps with a pedometer and increase the average number of steps by 30% within 7 days. At the same time, they should do two sets of calisthenics every day, each set including 15 leg raises, squats, and abdominal and back muscle strengthening exercises. | Control group (without exercise) | A two-year sports training program, conducted daily. |
| Iwamoto | 1998 | Japan | The training requires the subjects to use a pedometer to measure their daily steps and increase the average number of steps by 30% through outdoor walking within 7 days. At the same time, they should perform two sets of calisthenics training every day, each set including 15 repetitions of straight leg raises, squats, and abdominal and back muscle strengthening exercises. | Control group (without exercise) | A 12-month exercise training program, conducted daily. |
| Jamka | 2021 | Poland | Three 60-minute endurance training sessions per week, with heart rate maintained at 50% - 75% of maximum heart rate (HR max), to enhance cardiovascular endurance. Participants' heart rates are kept around 50% - 75% of their HR max. | Three 60-minute comprehensive training sessions per week, combining strength and endurance to enhance overall physical fitness. The training intensity is set at 50% - 60% of each person's maximum repetition capacity. | The 12-week program consists of 36 training sessions. Participants must complete at least 80% of them. They are required to attend three sessions per week, each lasting approximately 60 minutes. |
| Junior | 2018 | Brazil | 5 minutes of warm-up, 20 minutes of high-intensity interval jump training (including hip abduction and adduction jumps, a total of 20 sets, each set 30 seconds of all-out sprint, 30 seconds rest between sets), and 5 minutes of relaxation/cool-down exercises (slow walking and stretching). | Control group (without exercise) | The aquatic exercise program consists of three 30-minute sessions per week for 24 weeks. The weekly training sessions are conducted on three different non-consecutive days. |
| Jessup | 2003 | USA | The training consists of a 5-minute warm-up, strength training (gradually increasing from 50% of 1RM to 75%), weighted walking (gradually increasing the load to 10% of body weight), 30-45 minutes of walking training and balance training (walking along lines and around cones), and ends with a 5-minute relaxation. | Control group (without exercise) | Exercise for 60 to 90 minutes three times a week for 32 weeks. |
| Kemmler | 2010 | Germany | The training requirements include 20 minutes of warm-up/aerobic dance (keeping the heart rate at 70% to 85% of the maximum heart rate), 5 minutes of balance training, functional gymnastics and isometric strength training (1 to 3 sets, 10 to 15 movements per set, holding for 6 to 10 seconds), elastic band upper limb exercises (2 to 3 sets, 10 to 15 repetitions per set), and dynamic weighted leg exercises (2 sets, 8 repetitions per set). The intensity is based on the maximum effort level minus 2 repetitions, and the difficulty is gradually increased. | Control group (without exercise) | The program lasts for 18 months. The weekly exercise plan includes two 60-minute supervised group classes and two 20-minute home training sessions. Health care plan: It lasts for 18 months. A 60-minute low-frequency and low-intensity training session is conducted once a week for 10 weeks, followed by a 10-week break. |
| Kwon | 2008 | South Korea | The training includes aerobic exercise, resistance training, weight-bearing exercises and balance movements, lasting for 60 minutes. | Control group (without exercise) | 24 weeks of training, three times a week, 60 minutes each time. |
| Korpelainen | 2006 | Finland | The training requires women to complete 45 minutes of jumping and balancing exercises in a one-hour supervised class, including 15 minutes of warm-up, and to do 20 minutes of similar training at home every day. | Control group (without exercise) | For a total of 30 months, the women in the exercise group were required to attend a 6-month training course each year, with each training session lasting 1 hour. Participants were also asked to train at home for 20 minutes every day, with the training content similar to that in the supervised training courses. Each year, they participated in a 6-month program. |
| lai | 2013 | China | The WBV group received high-frequency (30 Hz) and high-amplitude (3.2g) WBV intervention for six months at the sports center in a natural standing position, for 5 minutes each time, three times a week. | Control group (without exercise) | For six months, five minutes each time, three times a week. |
| Li | 2023 | China | The training requirements include a 5-minute warm-up and cool-down, combining the Yi Jin Jing exercises with resistance training using elastic bands. The number of repetitions of the movements gradually increases, and the exercise duration gradually extends from 47 minutes to 76 minutes. The intensity is controlled at 60% - 70% of the maximum heart rate to ensure moderate-intensity exercise. | Control group (without exercise) | The exercise time (8:00 a.m. to 9:30 a.m. from Monday to Friday) and location are uniform and fixed. The exercise frequency is three times a week, with no more than two days between each training session, and it lasts for six months. |
| Lord | 1996 | Australia | The training lasts for one hour, including warm-up and adaptation period (aerobic exercise, balance and coordination training, and strength training). Among them, 35 to 40 minutes are spent on weight training, covering flexibility, muscle strengthening and coordination exercises for legs, trunk and arms. | Control group (without exercise) | Attend two 1-hour fitness classes each week for a total of four 10 to 12-week courses, with three 1 to 2-week breaks during the academic terms and one 5-week break in between - totaling 42 weeks of exercise. |
| Moreira | 2014 | Brazil | Each class lasts for 50 to 60 minutes, including 10 minutes of warm-up exercises, followed by strength/endurance training, then cardio training, and concludes with 10 minutes of stretching and balance training. | Control group (without exercise) | Attend three aquatic aerobics classes per week for 24 weeks, with each class lasting 50 to 60 minutes. |
| MARTIN | 1993 | USA | The training was conducted on an electric treadmill with adjustable slope, at an intensity of 70 - 85% of the maximum heart rate measured for each subject. At the beginning and end of each training session, a warm-up and cool-down period of 3 - 5 minutes was carried out at approximately 60% of the maximum heart rate. | Control group (without exercise) | The exercise training will last for 12 months, each session lasting 30 to 50 minutes, and it will be conducted three times a week. |
| Marques | 2010 | Portugal | Resistance training is conducted three times a week (not consecutively), each session lasting approximately 60 minutes. | The training consists of 10-15 minutes of warm-up, 35-40 minutes of dynamic aerobic exercise, 10 minutes of strength training and 10 minutes of relaxation. The intensity gradually increases from 50% to 60% of the heart rate reserve to 65% to 85%.  Control group (without exercise) | A 32-week endurance sports training program, with three training sessions per week, and at least one day of rest between each session. Each training session lasts approximately 60 minutes. |
| Marques | 2011 | Portugal | The training consists of 10 minutes of warm-up, 15 minutes of weight-bearing activities, 10 minutes of muscle endurance training, 10 minutes of balance training, 10 minutes of agility training and 5 minutes of stretching. The number of repetitions for weight-bearing and strength training has been increased from 8 to 15, and the number of sets has been increased to 3. | Control group (without exercise) | A 32-week progressive multi-component training program, with two sessions per week, each lasting approximately 60 minutes. |
| MOSTI | 2013 | Norway | The training requires the use of a lower body squat machine for squat exercises. Start with a warm-up (2 sets of 8-12 reps, with a load of 50%), followed by 4 sets of 3-5 reps, with a load of 85%-90% of 1RM, emphasizing the exertion of maximum force during the concentric phase. Take a 2-3 minute break between sets and adjust the load dynamically based on the participant's performance. | Control group (without exercise) | A 12-week training program, conducted three times a week for a total of 36 sessions, includes supervised muscle rapid strength training (MST). |
| Nicholson | 2014 | Australia | The exercises include squats, lunges and chest presses, etc. Use relatively light weights and do a large number of repetitions. The pre-arranged course is divided into 10 parts, each lasting about 6 minutes. | Control group (without exercise) | Twice a week for six months, each class lasts about 50 minutes. |
| Nambi | 2020 | Saudi Arabia | Virtual reality training requires participants to stand. | This exercise training program includes vertical jumps, aerobic exercise and graded walking: participants jump from a height of 5 cm for 10 times and gradually increase to 25 cm; after jumping, they do 30 minutes of general aerobic exercise; after the aerobic exercise, they walk within the range of 50% to 75% of their heart rate. "  Control group (without exercise) | Train for 45 minutes every day, 4 days a week, for 12 weeks. |
| Oliveira | 2018 | Brazil | Undergo 5 minutes of whole body vibration (WBV) treatment on a sinusoidal waveform vibration platform. | All exercises should be performed in one set of 10 repetitions, with a one-minute rest between each exercise.  Control group (without exercise) | Three times a week, with one day's interval each time, for six months. |
| Park | 2008 | Japan | Each session includes 9 minutes of stretching, 10 minutes of strength training, 23 minutes of weight-bearing exercises at an intensity of 65% to 75% of the maximum heart rate, and 18 minutes of balance and posture correction training. | Control group (without exercise) | The 48-week exercise group participated in a 48-week exercise program, three times a week. |
| Pruitt | 1995 | USA | Perform 3 sets of 14 repetitions each of resistance training, with similar training volume (weight × repetitions). Test 1-RM every 2 weeks for the first 3 months and then every 3 weeks to adjust the load. | Control group (without exercise) | Twelve months of resistance training of different intensities, twice a week. |
| Riaz | 2024 | Pakistan | Each single VRT training session lasts for 45 minutes and is divided into three stages: warm-up, activity and relaxation. It is based on the X-Box 360 Kinect game and is conducted three times a week for 24 weeks. Meanwhile, the experimental group needs to take a 30-minute outdoor walk every day. | The control group needs to take a 30-minute outdoor walk every day. | Three times a week for 24 weeks. |
| Rhodes | 2000 | Canada | The training requires a 20-minute warm-up (10 minutes of cycling + 10 minutes of easy stretching), followed by strength training. Complete each exercise in a circuit style, doing 3 sets of 8 repetitions for each. | Control group (without exercise) | A three-month strength training program (three times a week, one hour each time). |
| SMIDT | 1992 | USA | Progressive resistance training for the trunk muscles | Control group (without exercise) | A year-long progressive resistance training program for the trunk muscles, maintained three to four times a week. |
| Slatkovska | 2011 | Canada | Perform 20 minutes of whole-body vibration training at home every day. The training parameters are an acceleration of 0.3g and a frequency of either 90Hz or 30Hz. | Control group (without exercise) | For 12 consecutive months, I did 20 minutes of whole-body vibration training at home every day. |
| Song | 2021 | China | Perform half squats, deep squats with heel raises, and alternating single-leg squats on each side under vibration conditions (five sets of ten repetitions for each movement, with a 30-second rest between sets). | Control group (without exercise) | The cycle is 24 months, three times a week, 20 minutes each time. |
| Stengel | 2011 | Germany | The vibration group applied vibration for 6 minutes in each training session and gradually increased the frequency from 25Hz to 30Hz and then to 35Hz at 3 months and 6 months. | The family training programs are changed every six months to maintain the diversity and effectiveness of the training.  Control group (without exercise) | For 18 months, both training groups were required to conduct two controlled training sessions (each lasting 60 minutes) and two home training sessions (each lasting 20 minutes) every week. The vibration group applied vibration for 6 minutes each time during training, and the frequency gradually increased from 25 to 30 and then to 35 Hz at 3 and 6 months respectively. The home training items were changed every six months. |
| Santin | 2015 | Spain | Train twice a week on a vibration platform (with a frequency of 20 hertz and an amplitude of 2 millimeters). | Control group (without exercise) | Twice a week for eight months |
| Tolomio | 2010 | Italy | Each training session has a specific goal (such as enhancing muscle strength, endurance, balance and joint flexibility), lasting approximately 60 minutes, and includes both calisthenics and exercises using tools like dumbbells, resistance bands, step boards and fitness balls. | Control group (without exercise) | An 11-month specific multi-component dual-mode exercise program |
| Tartibian | 2011 | Iran | Walk or jog on a treadmill for 25 to 30 minutes each day. Do this 3 to 4 days a week, with each exercise session at 45 to 55% of your determined HRmax. In the second 12 weeks of the study, increase the exercise duration to 40 to 45 minutes each day. Do this 4 to 6 days a week, with an exercise intensity of 55% to 65% of your HRmax. | Control group (without exercise) | For a period of six months, 3 or 4 days per week. |
| Verschueren | 2004 | Belgium | The WBV group performed static and dynamic knee extensor training on a vibration platform (vibration frequency 35 - 40 Hz, acceleration 2.28 - 5.09g). | The RES group trained the knee extensors through dynamic leg push and leg extension exercises, with the training intensity gradually increasing from low (20 repetitions of maximum) to high (8 repetitions of maximum).  Control group (without exercise) | The whole body vibration (WBV) group and the resistance training (RES) group trained three times a week for 24 weeks. There were 72 training sessions within 24 weeks. The training frequency was three times a week, with at least one day of rest between each training session. |
| Wavell | 2001 | UK | Fast walking is required to be completed continuously for 280 minutes every two weeks throughout the year. | Control group (without exercise) | For a year, each walk should last at least 20 minutes and brisk walking should be done at least three times a week. |
| WAVELL | 1997 | Japan | The time each subject took to walk 1600 meters on a flat road was used to obtain the measurement value of brisk walking speed. The duration of exercise was specified by a target every two weeks, increasing from 120 minutes to 280 minutes in the first three months and remaining at 280 minutes thereafter. | Control group (without exercise) | The time each subject took to walk 1600 meters on a flat road was used to obtain the measurement value of brisk walking speed. The duration of exercise was specified by a target every two weeks, increasing from 120 minutes to 280 minutes in the first three months and remaining at 280 minutes thereafter. |
| Wen | 2016 | China | The exercise session lasts for 90 minutes, including 10 to 15 minutes of warm-up, 30 to 35 minutes of aerobics (reaching and maintaining the target heart rate), 10 to 15 minutes of balance and relaxation, and 10 to 15 minutes of stretching and relaxation. The movements cover traditional steps, alternating knee lifts, arm movements, etc., accompanied by music at 120 to 126 beats per minute, with a moderate to high intensity. | Control group (without exercise) | A 12-month high-intensity exercise program (two to three times a week) involving stepping and jumping. |
| Watson | 2018 | Australia | A single HiRIT training session lasts for 30 minutes and includes four basic movements (deadlift, overhead press, and squat) performed in 5 sets of 5 repetitions each, with an intensity of 80% to 85% of 1RM. Optionally, 2 sets of 50% to 70% of 1RM deadlifts can be used for warm-up, combined with shock loading through jump pull-ups with landing cushioning. The training is conducted in small groups (up to 8 people). | Control group (without exercise) | An 8-month high-intensity resistance training program, twice a week, 30 minutes each time. |
| Wang | 2015 | China | Each workout consists of a 10-minute warm-up, five sets of the "42-style competition" movements, each set lasting 6 minutes with a 2-minute break between sets, and a final 10-minute relaxation activity. | Each workout consists of a 10-minute warm-up, six sets of 5-minute exercises (with a 2-minute break between sets), and a final 10-minute relaxation activity.  Control group (without exercise) | Practice four times a week, 60 minutes each time, for 12 months. |
| Xiang_yan | 2008 | China | The subjects stood upright on the vibration platform, lightly holding the handrails with both hands, with their feet shoulder-width apart, their bodies straight, and their center of gravity shifted to the heels. They received 10 minutes of vibration treatment at a frequency of 30 Hz and an amplitude of 5 mm. Blood pressure was measured and recorded before and after the treatment. The treatment was conducted 5 times a week for 4 weeks as one course, and a total of 6 courses were completed. | Control group (without exercise) | A six-month vibration therapy program |
| Young | 2007 | Australia | Take a 45-minute line dance (LD) class once a week for a year. | Take a line dance class once a week and do five sets of gradually increasing-load squats each week for a year.  Take a line dance class once a week, do five sets of gradually increasing load squats every week, and do five sets of foot stomping exercises twice a day every week for a year. | Group 1, Group 2 and Group 3 all participated in a 45-minute line dance (LD) class once a week for a year. Group 2 and Group 3 also completed a home squat training program (LDS). Group 3 additionally performed left and right foot stomping exercises (LDSS) twice a day. This lasted for a year. |
| Yamazaki | 2004 | Japan | Walk for at least one hour every day and take more than 8,000 steps. | Control group (without exercise) | At least 4 days a week for 12 months. |
| Yu | 2019 | China | Participants attend three non-consecutive 60-minute sessions each week for 24 weeks. Each session begins with a 10-minute light warm-up (including aerobics and stretching exercises), followed by 35 minutes of core aerobic dance training. The session concludes with 10 to 15 minutes of relaxation activities. | Control group (without exercise) | A 24-week aerobic dance course, held three times a week. |
